# Medically Assisted Reproduction and Hormone-Related Cancers

**DOI:** 10.1001/jamanetworkopen.2026.22832

**Published:** 2026-07-13

**Authors:** Adrian Raymond Walker, Christos Venetis, Signe Opdahl, Antoinette C. Anazodo, Neville F. Hacker, Michael Chapman, Louisa Jorm, Robert J. Norman, Catharyn Stern, Ursula M. Sansom-Daly, Georgina Mary Chambers, Claire Melissa Vajdic

**Affiliations:** 1 Centre for Big Data Research in Health, University of New South Wales, Sydney, Australia; 2 Unit for Human Reproduction, First Department of Obstetrics/Gynecology, Medical School, Faculty of Health Sciences, Aristotle University of Thessaloniki, Thessaloniki, Greece; 3 Department of Public Health and Nursing, Faculty of Medicine and Health Sciences, Norwegian University of Science and Technology, Trondheim, Norway; 4 Kids Cancer Centre, Sydney Children’s Hospital, Randwick, Australia; 5 Women’s Health, Paediatrics & Child Health, School of Clinical Medicine, University of New South Wales, Sydney, Australia; 6 St George Hospital, School of Women’s and Children’s Health, University of New South Wales, Sydney, Australia; 7 Robinson Research Institute, Faculty of Health and Medical Sciences, Adelaide University, Adelaide, Australia; 8 Melbourne IVF Melbourne, Australia; 9 Behavioural Sciences Unit, Discipline of Paediatrics and Child Health, School of Clinical Medicine, Faculty of Medicine and Health, University of New South Wales, Randwick, Australia; 10 Kirby Institute, University of New South Wales, Sydney, Australia; 11 Department of Obstetrics, Gynaecology and Newborn Health, University of Melbourne, Melbourne, Australia

## Abstract

**Importance:**

It is critical that women undertaking medically assisted reproductive (MAR) treatment and their clinicians know whether the treatments are associated with an increased risk of hormone-related cancers.

**Objective:**

To determine the risk of hormone-related cancers following MAR treatment.

**Design, Setting, and Participants:**

This cohort study used an emulated target trial design including Australian health registries and administrative datasets. Participants included women enrolled in Medicare, Australia’s universal health insurance scheme, aged 18 to 55 years between January 1, 1991, and December 31, 2018. Data were analyzed from April 2024 to July 2025.

**Exposures:**

Exposures were defined from Medicare records: assisted reproduction therapy, intrauterine insemination or ovarian stimulation, and ovulation induction with clomiphene citrate.

**Main Outcomes and Measures:**

Hormone-related invasive cancers (identified in the Australian Cancer Database) included breast, ovarian, uterine, thyroid, colorectal cancers and melanoma; in situ cancers included breast cancer and melanoma. Three cancers with no hormonal links—pancreatic, lung, and hematological—were included as negative controls. Flexible parametric survival models ascertained hazard ratios (HRs) and cumulative marginal survival differences in incident cancers per 100 000 population. E-values assessed the risk of bias due to unmeasured confounding.

**Results:**

A total of 1 748 927 women were identified, including 396 661 with history of MAR exposure. Although elevated risk of most hormone-related cancers was observed after MAR treatment (HRs, 1.09-1.64), E-value analysis suggested confounding due to underlying infertility conditions (ie, endometriosis, polycystic ovarian syndrome) could account for this observed elevation for uterine, ovarian, and thyroid cancers. For any specific invasive cancer, fewer than 20 extra cancers per 100 000 women each year were estimated for treated vs comparator groups. Emulated trials on the 6 hormone-related cancers and pancreatic and hematological cancers showed increased cancer risk in the first years after treatment, suggesting detection bias. Increased risk of hematological cancers was observed after MAR treatment (HRs, 1.18-1.27), indicating uncontrolled confounding by race and ethnicity may account for observed excess risk seen for several cancers. Some treatments were associated with decreased lung cancer risk (HRs, 0.72-0.82).

**Conclusions and Relevance:**

In this cohort study of MAR and cancer using a target trial emulation design, although associations between MAR and some hormone-related cancers were observed, the estimated difference in the number of expected cancers was small and may be explained by unmeasured confounding and detection bias.

## Key Points

**Question:**

Is medically assisted reproduction treatment associated with an increased risk of hormone-related cancers?

**Findings:**

In this cohort study of 1 748 927 women in Australia aged 18 to 55 years, hormone-related cancers were higher after some types of medically assisted reproduction treatment. This excess may be partly or wholly due to detection bias and unmeasured confounding from infertility-related conditions, like endometriosis or polycystic ovary syndrome, and anovulation, obesity, and race and ethnicity.

**Meaning:**

These findings suggest clinicians and women seeking medically assisted reproduction should be aware that hormone-related cancer risk could be associated with treatment, although this risk may not be attributable to the treatment itself.

## Teaser

This cohort study uses an emulated target trial design to evaluate the risk of hormone-related cancers after medically assisted reproductive treatment.

## Introduction

Medically assisted reproduction (MAR) refers to a collection of treatments that assist individuals to achieve a successful pregnancy. MAR treatments involving the extraction and in vitro handling of ova (eggs) are referred to as *assisted reproductive technologies* (ART). These treatments are generally conducted with medical stimulation of the ovaries to retrieve multiple ova in a single menstrual cycle (ie, ovarian stimulation [OS]). These eggs are fertilized through in vitro fertilization, either by mixing the sperm with the egg, or intracytoplasmic sperm injection. MAR treatments without in vitro handling of ova include ovulation induction through hormonal medications or by intrauterine insemination (IUI), in which prepared sperm is inserted into the uterus around the time of ovulation. MAR treatments have become increasingly common, with approximately 3 million ART cycles performed each year worldwide.^1^

In most cases, MAR therapies involve the administration of medications that are either hormonal or hormone-modulating agents, including gonadotrophin-releasing hormone analogues, follicle stimulating hormone, human chorionic gonadotrophin, progesterone, estrogen, and clomiphene citrate or letrozole. There has long been concern that these medicines might be associated with the development of hormone-related cancers, including ovarian, uterine, breast, melanoma, thyroid, and possibly colorectal cancers.^2,3,4,5^ In ART treatment, it is also possible the repeated puncture of follicles during ovum retrieval might cause breakdown and repair of the ovarian surface epithelium similar to that in natural ovulations, consistent with the incessant ovulation hypothesis for ovarian carcinogenesis.^6,7^

Despite the biological plausibility of these hypotheses,^6,8,9,10,11^ strong evidence for any carcinogenic effect of MAR medicines is limited.^12,13^ Due to the rarity of some cancers, the long latency period for most solid cancers, and ethical concerns around withholding treatment for infertility, randomized clinical trials cannot be used to examine this question.^12^ Recent meta-analyses on observational studies suggest few to no associations of ART with hormone-related cancer risk in female patients.^14,15,16^ However, there is substantial heterogeneity in study designs and availability of confounding variables. This heterogeneity is compounded by changes in patterns of MAR use over time and between-country differences in eligibility for and access to MAR.

In general, most studies conducted since 2010 adjusted for relevant confounders, like parity, maternal age, and socioeconomic status.^14^ When meta-analyzing only these studies, no significant associations of MAR treatment with breast, ovarian, or uterine cancer were found.^14^ Considering specific studies reported over the past 10 years, Vassard et al^17^ reported a significant association between ART and invasive breast cancer, and Lundberg et al^18^ reported a heightened risk of ovarian cancer following ART in individuals who gave birth following treatment. Kessous et al^19^ reported high excess risks for both ovarian and uterine cancer but no increased risk for breast cancer (combining in situ and invasive). Reigstad et al^20^ found increased risk following ART treatment for thyroid cancer, but not other types of hormone-related cancer (noting wide CIs). However, Reigstad et al^20^ did find increased risks of uterine cancer following use of clomiphene citrate, invasive breast cancer following clomiphene citrate for parous individuals, and ovarian cancer for nulliparous individuals. Spaan et al^21^ found evidence that borderline ovarian cancers, but not invasive ovarian cancers, were more common following ART. Overall, there is substantial variability in both confounder adjustment and analysis approach, with this variability contributing to heterogenous results.

Understanding the risks after undertaking MAR treatments is critical for female patients and their health care practitioners.^14,15,16,22^ This study aims to untangle the complex relationship between MAR and hormone-related cancers using the emulated target trial framework.^23^ To our knowledge, no studies have leveraged the target trial framework to analyze the association of MAR with cancer. We also include bias assessment (through calculation of E-values),^24^ negative controls, and assessment for time-varying treatment effects. In doing so, we build on previous work by directly assessing the likely impact of missing confounders, and the likelihood that detection bias might contribute to reported associations between MAR and cancer.

## Methods

This cohort study used a retrospective emulated target trial design and was conducted as part of a broader body of work on cancer after MAR in Australia.^25^ The methods are detailed fully in the eMethods in Supplement 1. The study was approved by all relevant human research ethics committees, including the Australian Institute of Health and Welfare human research ethics committees. Data were accessed and used under a waiver of informed consent, and researchers were granted access only to linked anonymized data. Our reporting follows the Transparent Reporting of Observational Studies Emulating a Target trial (TARGET) guidelines for reporting of emulated target trials.^26,27^ The ideal randomized trial for this study is described in eTable 1 in Supplement 1.

Briefly, we conducted a linkage of Australian state and national data to identify women who received MAR between January 1, 1991 and December 31, 2018. A description of the datasets used is given in eTable 2 in Supplement 1. While the term *women* is used, this study includes all people assigned female at birth, including gender diverse and nonbinary people. Women who received MAR were identified by the Australian Institute of Health and Welfare as those who had a record of reimbursement for a MAR treatment on the Medicare Benefits Scheme (Australia’s national public health insurance scheme) or of reimbursement for a relevant MAR medication on the Pharmaceutical Benefits Scheme (Australia’s national medication subsidization program) (eTable 3 in Supplement 1). The Australian Institute of Health and Welfare then identified matched women who were never exposed to MAR (up to 4:1) and made the anonymized cohorts available to the research team. Women were matched on birth year, residential remoteness (metropolitan or rural), parity at matched exposed individual’s first MAR exposure date, and (if the matched exposed individual was parous at their first MAR) their age at their first birth (±365 days). Due to an error in data extraction, approximately 300 000 comparator women identified by the AIHW for study inclusion were not in the country at the beginning of analysis and had to be excluded, as we could not determine the presence or absence of cancer. This error created an imbalance in the number of nulliparous women in the MAR treatment and comparator groups, which was accounted for later through inverse probability of treatment weighting.

eFigure 1 in Supplement 1 shows the steps taken to ascertain the cohort of eligible women for treatment strategy assignment. Three types of MAR treatment were identified by the presence of relevant procedural or prescription codes (eTable 4 in Supplement 1) in the first 28 days following first MAR exposure of any type: ART treatments (in vitro fertilization or intracytoplasmic sperm injection); IUI or OS with follicle stimulating hormone or ART cancelled before egg retrieval; and ovulation induction using clomiphene citrate (from 2002 only).

This first 28-day period from first MAR treatment was defined as time zero (t0), with study-time partitioned into 28-day blocks from this point forward. Women could contribute to multiple treatment groups if they had the relevant treatment codes, although this was uncommon (<10% of included women).

Outcomes included female cancers with sufficient evidence of an association with hormonal agents, specifically breast (invasive and in situ, separately), ovarian (serous and nonserous, separately and combined), and uterine.^28^ Melanoma (invasive and in situ), colorectal cancer, and thyroid cancer were also included because hormones may be relevant to their development.^29,30,31,32^ Cancers not considered hormone related (pancreatic, lung, and hematological) were included as negative controls. Only invasive tumors were considered (*International Classification of Diseases for Oncology, 3rd Edition* [*ICD-O-3*] behavior code 3), unless specified. All cancers were classified using *ICD-O-3* topographies and morphologies in the Australian Cancer Database (eTable 5 in Supplement 1).

### Statistical Analysis

Following calculation of descriptive statistics, we conducted an emulated target trial using flexible parametric survival modeling, setting α = .05 as statistically significant. We controlled for confounding using standardized inverse probability of treatment weighting. Confounding variables included age at t0, history of giving birth prior to t0, residential remoteness at t0 (metropolitan, inner regional, remote, or very remote), mean Index of Relative Socioeconomic Disadvantage percentile for area of residence, subsidized diabetes medication record prior to t0, and cancer registry notified cancer diagnosis prior to t0. Exposures were the 3 different MAR exposures, and outcomes were all hormone-related and negative control cancers. We tested for time-varying associations with treatment using likelihood ratio tests comparing 4 models: a baseline model with no time-varying association of treatment and 3 models with time-varying associations with differing numbers of splines in the hazard function (0, 1, or 3 splines).

Women were followed up until either a relevant cancer outcome, December 31, 2019, or death (as recorded in the National Death Index), whichever occurred first. We report hazard ratios (HRs) and cumulative marginal survival difference per 100 000 population in expected cancer diagnoses.

We estimated how much uncontrolled confounding would be needed to account for our observed associations using E-values.^24^ E-values are the minimum association (on the risk ratio scale) an uncontrolled confounder would need to have between both the exposure and outcome to fully explain the observed association. For time-varying HRs, E-values are given at year 1, and then every 5 years starting at year 5.

Data were analyzed using SAS version 9 (SAS Institute) and Stata version 18 (StataCorp) from April 2024 to July 2025.

## Results

We identified 1 748 927 women, including 396 661 women exposed to MAR (Table 1). During follow-up for cancer, 0.4% to 1.3% of the cohorts were censored at death; 4.0% of women who received MAR identified by the Australian Institute of Health and Welfare were not eligible for inclusion due to missing data on state (or state recorded as Northern Territory), sociodemographic area of residence, or remoteness at t0. We assumed these women were missing at random and proceeded with complete-case analysis. eTable 6 and eTable 7 in Supplement 1 show the total number of incident cancers and the results of the model selection steps respectively.

**Table 1.** Demographic Profile of the 3 Treatment and 3 Comparator Cohorts

Characteristic	Women, No. (%)^a^					
	ART treatment		IUI or OS treatment		Clomiphene citrate dispensation	
	Treatment	Comparator	Treatment	Comparator	Treatment	Comparator
Overall	171 609 (22.7)	584 311 (77.3)	77 767 (23.0)	260 280 (77.0)	156 084 (22.5)	537 796 (77.5)
Age at study entry, y						
Mean (SD)	34.669 (5.054)	34.851 (5.022)	34.019 (5.143)	34.233 (5.116)	31.766 (5.434)	32.008 (5.442)
Median (IQR)	35 (31-38)	35 (31-39)	34 (30-38)	34 (31-38)	31 (28-35)	32 (28-35)
Year at t0						
1991-1994	12 540 (7.3)	37 985 (6.5)	8486 (10.9)	25 621 (9.8)	NA	NA
1995-1999	17 139 (10.0)	54 633 (9.3)	13 438 (17.3)	42 572 (16.4)	NA	NA
2000-2004	22 464 (13.1)	75 113 (12.9)	13 185 (17.0)	43 654 (16.8)	25 671 (16.4)	86 458 (16.1)
2005-2009	36 233 (21.1)	126 562 (21.7)	13 854 (17.8)	48 365 (18.6)	47 008 (30.1)	162 948 (30.3)
2010-2014	43 925 (25.6)	151 406 (25.9)	16 002 (20.6)	55 099 (21.2)	51 456 (33.0)	175 471 (32.6)
2015-2018	39 308 (22.9)	138 612 (23.7)	12 802 (16.5)	44 969 (17.3)	31 949 (20.5)	112 919 (21.0)
State						
Australian Capital Territory	2861 (1.7)	10 042 (1.7)	2144 (2.8)	4659 (1.8)	2591 (1.7)	9272 (1.7)
New South Wales	62 546 (36.4)	197 861 (33.9)	24 527 (31.5)	90 755 (34.9)	53 463 (34.3)	178 278 (33.1)
Queensland	26 869 (15.7)	110 667 (18.9)	20 631 (26.5)	47 510 (18.3)	33 378 (21.4)	104 871 (19.5)
South Australia	12 779 (7.4)	35 766 (6.1)	4287 (5.5)	15 806 (6.1)	9263 (5.9)	35 336 (6.6)
Tasmania	2985 (1.7)	11 154 (1.9)	1673 (2.2)	4994 (1.9)	2707 (1.7)	10 996 (2.0)
Victoria	48 565 (28.3)	161 767 (27.7)	15 910 (20.5)	71 824 (27.6)	45 598 (29.2)	143 750 (26.7)
Western Australia	15 004 (8.7)	57 054 (9.8)	8595 (11.1)	24 732 (9.5)	9084 (5.8)	55 293 (10.3)
Remoteness						
Major cities	141 187 (82.3)	463 758 (79.4)	64 901 (83.5)	207 410 (79.7)	121 390 (77.8)	418 872 (77.9)
Inner regional	20 091 (11.7)	82 804 (14.2)	9201 (11.8)	37 701 (14.5)	23 064 (14.8)	78 463 (14.6)
Outer regional	8544 (5.0)	31 639 (5.4)	3213 (4.1)	12 683 (4.9)	9740 (6.2)	33 592 (6.2)
Remote	1234 (0.7)	3946 (0.7)	317 (0.4)	1636 (0.6)	1379 (0.9)	4492 (0.8)
Very remote	553 (0.3)	2164 (0.4)	135 (0.2)	850 (0.3)	511 (0.3)	2377 (0.4)
Area-based Index of Relative Socioeconomic Disadvantage quintile						
1 (Most disadvantaged)	20 996 (12.2)	86 587 (14.8)	9191 (11.8)	38 582 (14.8)	26 832 (17.2)	83 173 (15.5)
2	22 700 (13.2)	88 645 (15.2)	9724 (12.5)	39 248 (15.1)	24 525 (15.7)	85 919 (16.0)
3	31 465 (18.3)	110 299 (18.9)	14 638 (18.8)	48 816 (18.8)	31 876 (20.4)	105 123 (19.5)
4	37 234 (21.7)	123 099 (21.1)	18 029 (23.2)	54 531 (21.0)	32 462 (20.8)	112 598 (20.9)
5 (Least disadvantaged)	59 214 (34.5)	175 681 (30.1)	26 185 (33.7)	79 103 (30.4)	40 389 (25.9)	150 983 (28.1)
Estimated parity at t0						
0	138 542 (80.7)	420 180 (71.9)	62 704 (80.6)	190 969 (73.4)	107 942 (69.2)	339 679 (63.2)
1	20 649 (12.0)	70 216 (12.0)	10 853 (14.0)	32 053 (12.3)	34 391 (22.0)	97 530 (18.1)
2	7363 (4.3)	54 922 (9.4)	2813 (3.6)	21 946 (8.4)	9100 (5.8)	62 385 (11.6)
3	3182 (1.9)	23 990 (4.1)	898 (1.2)	9500 (3.6)	2880 (1.8)	24 809 (4.6)
4	1253 (0.7)	9046 (1.5)	308 (0.4)	3478 (1.3)	1020 (0.7)	8685 (1.6)
≥5	620 (0.4)	5957 (1.0)	191 (0.2)	2334 (0.9)	751 (0.5)	4708 (0.9)
History of diabetes at t0						
Yes	3876 (2.3)	8431 (1.4)	1583 (2.0)	2893 (1.1)	10 776 (6.9)	8109 (1.5)
ART^a^						
At t0	171 609 (100.0)	NA	1715 (2.2)	NA	309 (0.2)	NA
Total treatments, No.	510 169	NA	32 052	NA	18 221	NA
IUI or OS^b^						
At t0	1715 (1.0)	NA	77 767 (100.0)	NA	6797 (4.4)	NA
Total treatments, No.	161 166	NA	213 646	NA	29 760	NA
Clomiphene citrate^b^						
At t0	309 (0.2)	NA	6797 (8.7)	NA	156 084 (100.0)	NA
Total treatments, No.	150 197	NA	68 023	NA	439 388	NA
Years in study						
Mean (SD)	11.083 (7.473)	10.781 (7.319)	13.439 (7.894)	13.046 (7.807)	9.083 (4.654)	9.029 (4.665)
Median (IQR)	10 (5-16)	10 (5-16)	13 (6-20)	12 (6, 20)	9 (5-13)	9 (5-13)
Died in study period	1512 (0.9)	5824 (1.0)	777 (1.0)	3318 (1.3)	627 (0.4)	2610 (0.5)

Abbreviations: ART, assisted reproductive technology; IUI, intrauterine insemination; NA, not applicable; OS, ovarian stimulation; t0, time zero.

^a^ Percentages are by column, except for overall, which is given as percentage across treatment type.

^b^ When approximating the number of treatments, only 1 treatment could occur in each 28-day analysis window.

**Figure 1.**
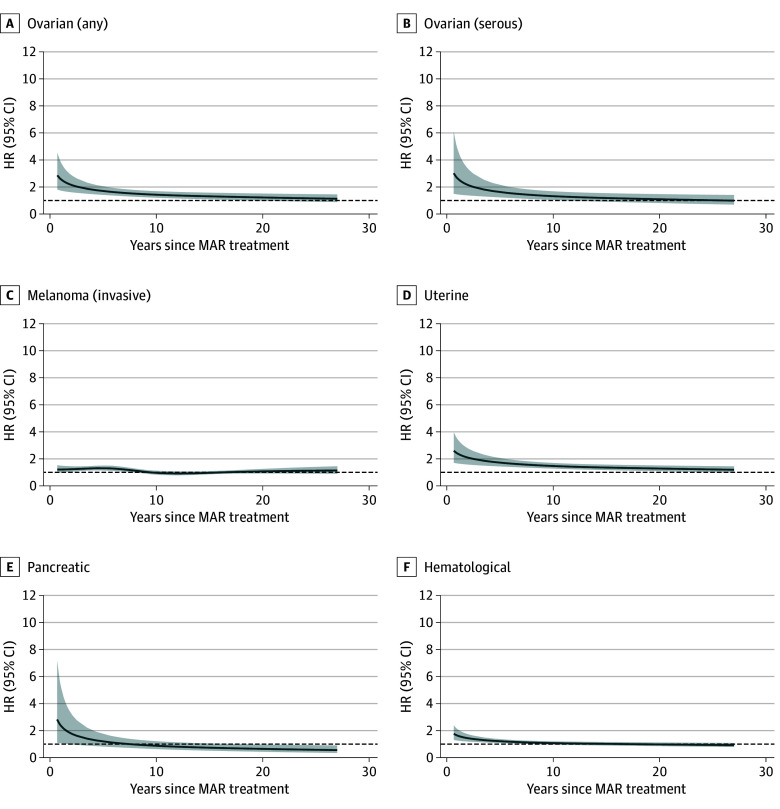
Line Graphs of Time-Varying Hazard Ratios for Associations of Any Assisted Reproduction Technology With Cancers Reported from 6 months. MAR indicates medically assisted reproduction.

**Figure 2.**
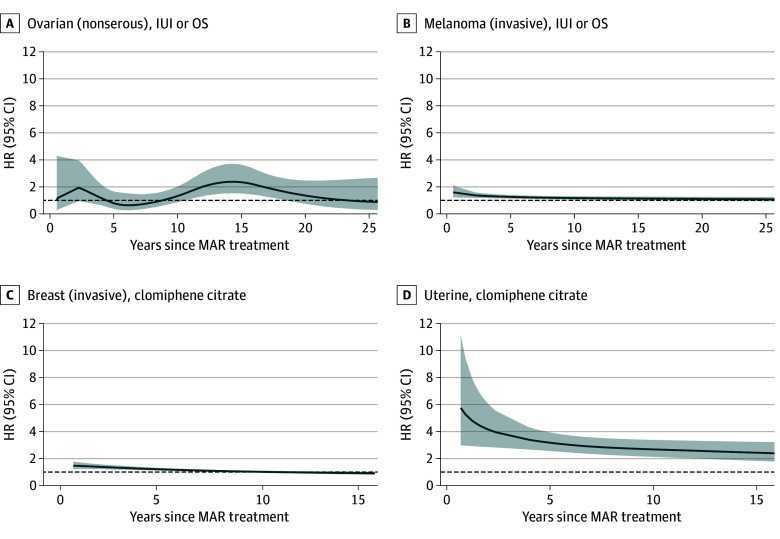
Line Graphs of Time-Varying Hazard Ratios for Associations of Intrauterine Insemination (IUI) or Ovarian Stimulation (OS) or Clomiphene Citrate With Cancer Reported from 6 months. MAR indicates medically assisted reproduction.

A summary of all modeling results is given in Table 2. Eight emulated trials of hormone-related cancers showed evidence of a time-varying HR. The most common pattern was an association of MAR with higher risk of cancer immediately after first treatment that decreased over time, reducing to a small or no difference in risk after approximately 10 years (Figure 1 and Figure 2). This was the case for any ovarian cancer, serous ovarian cancer (47% of ovarian cancers), uterine cancer following ART, invasive melanoma following IUI or OS, and invasive breast and uterine cancer following clomiphene citrate (Figure 2). The observed association with an excess cancer risk was maintained across the follow-up period for only uterine cancer following clomiphene citrate (Figure 2). ART was associated with increased risk for invasive melanoma, and IUI or OS was associated with increased risk of nonserous ovarian cancer up to approximately 5 years after treatment and then were associated with decreased risk, reaching no association with excess risk before 10 years after treatment.

**Table 2.** Summary Table of Results

MAR treatment type	Cancers with time-varying hazard ratio	Cancers with non-time-varying increased hazard ratio	Range^a^	
			Hazard ratios	E values
ART	Ovarian (any), ovarian (serous), melanoma (invasive), pancreatic, haematological	Breast (invasive and in situ), ovarian (nonserous), uterine, thyroid, colorectal, melanoma (in situ)	1.11-1.64	1.45-2.66
IUI or OS	Ovarian (nonserous), melanoma (invasive)	Breast (invasive), ovarian (any), ovarian (serous), uterine, melanoma (in-situ), hematological	1.09-1.54	1.40-2.45
Clomiphene Citrate	Breast (invasive), uterine	Ovarian (any), ovarian (nonserous), thyroid, melanoma (both invasive and in situ), hematological	1.18-1.42	1.65-2.19

Abbreviations: ART, assisted reproductive technology; IUI, intrauterine insemination; MAR, medically assisted reproduction; OS, ovarian stimulation.

^a^ Calculated from central estimate for cancers with a significant effect (α = 0.05) without a time-varying hazard ratio.

In trials where the HRs were not observed to vary over time, all types of MAR treatment were associated with elevated risks of most cancers, with HR estimates ranging from 1.09 (95% CI, 1.03-1.16) (invasive breast cancer following IUI or OS) to 1.64 (95% CI, 1.31-2.05) (nonserous ovarian cancer following ART) (Table 3). There was no elevation in risk for in situ breast, thyroid, or colorectal cancer following IUI or OS or in situ breast, serous ovarian, or colorectal cancer following clomiphene citrate use.

**Table 3.** Hazard Ratios, E Values, and Cumulative Marginal Survival Difference in Incident Cancers for Each Emulated Target Trial

MAR treatment type	HR			Cumulative marginal survival difference in incident cancers per 100 000 population					
	Estimate (95% CI)	SE	E value (95% CI)	1 y	5 y	10 y	15 y	20 y	25 y
**ART**									
Breast (invasive)	1.11 (1.06 to 1.16)	0.03	1.45 (1.30 to 1.59)	3 (2 to 5)	31 (16 to 45)	94 (51 to 138)	187 (101 to 274)	303 (163 to 443)	438 (236 to 640)
Breast (in situ)	1.30 (1.10 to 1.52)	0.11	1.91 (1.44 to 2.42)	1 (0 to 2)	12 (4 to 19)	43 (14 to 71)	98 (32 to 164)	180 (58 to 301)	286 (90 to 482)
Ovarian	Time varying	Time varying	Time varying	5 (2 to 7)	19 (11 to 27)	5 (2 to 7)	19 (11 to 27)	36 (22 to 51)	54 (31 to 77)
Ovarian (serous)	Time varying	Time varying	Time varying	2 (0 to 3)	8 (3 to 13)	2 (0 to 3)	8 (3 to 13)	15 (5 to 24)	21 (5 to 37)
Ovarian (nonserous)	1.64 (1.31 to 2.05)	0.19	2.66 (1.94 to 3.52)	1 (0 to 2)	7 (3 to 11)	17 (8 to 27)	32 (15 to 49)	53 (26 to 80)	79 (38 to 120)
Uterine	Time varying	Time varying	Time varying	4 (2 to 6)	16 (9 to 24)	4 (2 to 6)	16 (9 to 24)	37 (23 to 51)	64 (40 to 88)
Thyroid	1.16 (1.04 to 1.29)	0.06	1.58 (1.23 to 1.90)	2 (0 to 3)	13 (3 to 22)	28 (6 to 50)	47 (10 to 84)	69 (15 to 123)	94 (21 to 167)
Colorectal	1.12 (1.00 to 1.25)	0.06	1.48 (1.02 to 1.80)	1 (0 to 2)	6 (0 to 12)	16 (0 to 33)	32 (−1 to 65)	56 (−1 to 114)	90 (−2 to 182)
Melanoma (invasive)	Time varying	Time varying	Time varying	6 (−2 to 13)	39 (16 to 62)	6 (−2 to 13)	39 (16 to 62)	67 (29 to 105)	51 (−4 to 107)
Melanoma (in situ)	1.14 (1.03 to 1.27)	0.06	1.54 (1.19 to 1.85)	4 (1 to 7)	24 (4 to 44)	66 (11 to 120)	138 (24 to 252)	235 (40 to 430)	353 (59 to 648)
Pancreatic	Time varying	Time varying	Time varying	1 (0 to 3)	3 (−1 to 7)	1 (0 to 3)	3 (−1 to 7)	3 (−4 to 10)	−1 (−13 to 10)
Lung	0.82 (0.69 to 0.97)	0.07	NA^a^	0 (−1 to 0)	−3 (−5 to 0)	−10 (−18 to −2)	−23 (−42 to −4)	−48 (−86 to −9)	−91 (−165 to −17)
Hematological	Time varying	Time varying	Time varying	6 (2 to 10)	22 (9 to 34)	6 (2 to 10)	22 (9 to 34)	33 (12 to 53)	38 (5 to 71)
**IUI or OS**									
Breast (invasive)	1.09 (1.03 to 1.16)	0.03	1.40 (1.19 to 1.59)	3 (1 to 5)	24 (7 to 42)	75 (21 to 128)	151 (43 to 258)	246 (71 to 422)	358 (103 to 613)
Breast (in situ)	1.16 (0.90 to 1.51)	0.15	NA^a^	1 (−1 to 2)	8 (−6 to 21)	24 (−19 to 66)	49 (−38 to 136)	83 (−64 to 230)	125 (−96 to 346)
Ovarian	1.48 (1.20 to 1.84)	0.16	2.33 (1.68 to 3.08)	1 (0 to 3)	9 (3 to 15)	25 (10 to 41)	48 (19 to 78)	81 (32 to 130)	125 (50 to 200)
Ovarian (serous)	1.49 (1.10 to 2.04)	0.24	2.35 (1.42 to 3.49)	0 (0 to 1)	3 (0 to 6)	10 (1 to 18)	21 (3 to 39)	40 (6 to 74)	69 (10 to 127)
Ovarian (nonserous)	Time varying	Time varying	Time varying	0 (−3 to 3)	6 (−5 to 17)	0 (−3 to 3)	6 (−5 to 17)	4 (−12 to 19)	29 (3 to 56)
Uterine	1.54 (1.29 to 1.84)	0.14	2.45 (1.90 to 3.09)	1 (0 to 2)	9 (4 to 13)	28 (15 to 42)	63 (33 to 93)	124 (66 to 181)	223 (120 to 325)
Thyroid	1.11 (0.95 to 1.29)	0.09	NA^a^	1 (−1 to 3)	8 (−4 to 21)	19 (−10 to 48)	31 (−16 to 79)	46 (−23 to 114)	61 (−32 to 154)
Colorectal	0.99 (0.86 to 1.15)	0.08	NA^a^	0 (−1 to 1)	0 (−8 to 7)	−1 (−22 to 20)	−2 (−41 to 38)	−3 (−73 to 67)	−4 (−118 to 109)
Melanoma (invasive)	Time varying	Time varying	Time varying	17 (6 to 28)	61 (29 to 92)	17 (6 to 28)	61 (29 to 92)	99 (52 to 147)	135 (66 to 204)
Melanoma (in situ)	1.39 (1.19 to 1.64)	0.11	2.14 (1.66 to 2.66)	7 (3 to 11)	59 (28 to 91)	173 (81 to 264)	352 (165 to 538)	584 (269 to 900)	864 (389 to 1340)
Pancreatic	0.85 (0.57 to 1.27)	0.17	NA^a^	0 (0 to 0)	−1 (−3 to 1)	−2 (−8 to 3)	−5 (−18 to 7)	−11 (−36 to 15)	−21 (−70 to 29)
Lung	0.72 (0.57 to 0.91)	0.09	NA^a^	0 (−1 to 0)	−4 (−7 to −1)	−14 (−24 to −5)	−33 (−55 to −11)	−68 (−112 to −23)	−130 (−215 to −45)
Hematological	1.27 (1.11 to 1.46)	0.09	1.86 (1.45 to 2.28)	3 (1 to 4)	16 (6 to 26)	38 (14 to 62)	69 (27 to 112)	119 (46 to 192)	190 (73 to 308)
**Clomiphene Citrate**									
Breast (invasive)	Time varying	Time varying	Time varying	9 (2 to 17)	64 (37 to 91)	104 (51 to 157)	64 (−45 to 174)	NA	NA
Breast (in situ)	1.15 (0.97 to 1.38)	0.10	NA^a^	0 (0 to 1)	3 (−1 to 8)	14 (−4 to 31)	34 (−9 to 76)	NA	NA
Ovarian	1.40 (1.07 to 1.81)	0.19	2.14 (1.36 to 3.03)	1 (0 to 2)	7 (1 to 13)	16 (2 to 29)	27 (4 to 50)	NA	NA
Ovarian (serous)	1.36 (0.90 to 2.06)	0.29	NA^a^	0 (0 to 1)	2 (−1 to 5)	6 (−3 to 14)	11 (−5 to 26)	NA	NA
Ovarian (nonserous)	1.42 (1.01 to 1.99)	0.24	2.19 (1.12 to 3.38)	1 (0 to 2)	5 (0 to 10)	10 (−1 to 20)	16 (−1 to 34)	NA	NA
Uterine	Time varying	Time varying	Time varying	6 (3 to 9)	33 (23 to 43)	75 (58 to 93)	144 (107 to 180)	NA	NA
Thyroid	1.30 (1.15 to 1.46)	0.08	1.91 (1.55 to 2.29)	3 (1 to 5)	24 (12 to 36)	58 (29 to 88)	96 (47 to 144)	NA	NA
Colorectal	1.15 (0.99 to 1.35)	0.09	NA^a^	1 (0 to 2)	7 (−1 to 16)	19 (−2 to 40)	37 (−4 to 79)	NA	NA
Melanoma (invasive)	1.22 (1.10 to 1.34)	0.06	1.73 (1.44 to 2.02)	4 (2 to 7)	29 (14 to 44)	67 (32 to 103)	120 (57 to 183)	NA	NA
Melanoma (in situ)	1.29 (1.17 to 1.42)	0.06	1.91 (1.63 to 2.20)	5 (3 to 7)	37 (22 to 51)	106 (63 to 149)	237 (142 to 333)	NA	NA
Pancreatic	0.96 (0.57 to 1.60)	0.25	NA^a^	0 (0 to 0)	0 (−2 to 1)	−1 (−6 to 5)	−1 (−15 to 13)	NA	NA
Lung	0.95 (0.72 to 1.26)	0.14	NA^a^	0 (0 to 0)	−1 (−4 to 3)	−2 (−11 to 8)	−5 (−31 to 22)	NA	NA
Hematological cancer	1.18 (1.02 to 1.37)	0.09	1.65 (1.18 to 2.07)	2 (0 to 3)	10 (1 to 20)	26 (2 to 49)	47 (5 to 90)	NA	NA

Abbreviations: ART, assisted reproductive technology; HR, hazard ratio; IUI, intrauterine insemination; MAR, medically assisted reproduction; NA, not applicable; OS, ovarian stimulation.

^a^ E values were not calculated for results where the HR’s 95% CI was entirely less than or included 1.

Regarding the negative control cancers, pancreatic cancer showed no increased risk after IUI or OS or after clomiphene citrate and had an increase in risk following ART. This association with ART was time varying, observed in the 5 years after treatment, then decreasing over time, a pattern that mirrored most hormone-related cancers with time-varying HRs. A similar pattern was observed for hematological cancers following all 3 MAR treatments. ART and IUI or OS were associated with decreased risk of lung cancer, and there was no association of lung cancer with clomiphene citrate.

Most treatments were estimated to be associated with fewer than 10 extra incident cases per 100 000 population per year, with only women treated with ART or IUI or OS approaching nearly 20 extra invasive breast cancers per 100 000 population per year (Table 3; eTable 8 in Supplement 1). E-value analysis indicated the association between the treatment and cancer outcome could be explained by a moderately strong confounder (risk ratio, 2) at the lower-bound CI (taken at the effect after 5 years for trials with time-varying hazard ratios (eTable 9 in Supplement 1), with the exception of uterine cancer after both ART and clomiphene citrate use.

We conducted a sensitivity analysis removing all women who had a reportable cancer (both invasive and in situ breast cancer and melanoma) prior to t0 (eTables 10-12 and eFigure 2 in Supplement 1). No major differences were observed that would substantially alter our conclusions.

## Discussion

In this cohort study applying an emulated target trial design to a cohort derived from national high-quality population-based datasets, we found MAR treatment was associated with an increase in risk of some hormone-related cancers. However, E-values and analysis of control non–hormone-related cancers suggested at least some of the increased risk was due to uncontrolled confounding and, for some cancers, bias due to differences in access to health care closer to the time of first treatment. The detection of such biases shows we did not emulate randomization sufficiently to emulate a target trial examining the impact of MAR on hormone-related cancers. However, we were able to leverage this framework to interrogate long-standing concerns with bias in studies on MAR and cancer. We also established that even with uncontrolled confounding, the absolute number of extra cancers expected following MAR treatment was relatively small.

### Cancer Risk Over Time and Detection Bias

Understanding the pattern of cancer risk over time since MAR is important, as there has long been concern that the observed associations between MAR and cancer may be biased by medical surveillance either while undergoing treatment or while pregnant.^33,34^ In our study, several emulated trials suggested a greater likelihood of incident cancer shortly after first treatment. This pattern suggests 2 possibilities: (1) MAR treatment (alone or in combination with pregnancy) promotes the growth of existing but undetected cancers or transformation of benign to invasive cancers or (2) there is increased surveillance during MAR treatment (and pregnancy), which leads to earlier detection of incident cancers (detection bias).

Overall, although it is possible specific MAR treatments are associated with cancer progression, it is difficult to reconcile this explanation with our observed risk pattern for 2 of the 3 negative control cancers. Therefore, we believe detection bias is the more likely explanation for these results. This finding is the first empirical indication using a negative control that medical surveillance may be responsible for an increased risk of cancer following MAR. This conclusion echoes that of Vassard et al,^33^ who observed a similar pattern in ovarian cancer risk after MAR using stratified Cox regression.

In Australia, individuals are invited to attend breast and colorectal cancer screening, and there is opportunistic skin cancer screening for individuals at high risk. According to Andersen’s behavioral model for use of health services,^35^ it is likely individuals who have engaged in fertility treatments are more likely to engage with these screening programs, and this disparity in seeking health services may also have affected our findings.

### Unmeasured Confounding

Our E-value analysis suggested some of the associations we observed could be explained by uncontrolled confounding. One such factor is the underlying cause of infertility, which was not recorded in any of the available datasets. Recent meta-analyses suggested endometriosis is associated with both any ovarian cancer (risk ratio, 1.93—noting nonserous ovarian cancer after ART had a lower-bound E-value of 1.94) and thyroid cancer (risk ratio, 1.39), which may explain our observed results for these cancers following ART and IUI or OS.^36,37^ The risk ratio for infertility for individuals with endometriosis compared with the general population has been estimated at 2.12,^38^ indicating for these cancers both the confounder-exposure and confounder-outcome relationship may have sufficient strength to account for the associations we observed.

Polycystic ovarian syndrome (PCOS) may also contribute to unmeasured confounding, particularly for uterine cancers and for clomiphene citrate users. Although it is uncommon to use clomiphene citrate for individuals with endometriosis-induced infertility (as endometriosis rarely causes anovulation), PCOS is partly defined by the presence of irregular ovulation,^39^ and clomiphene citrate is commonly prescribed for individuals with PCOS-induced infertility. A 2023 meta-analysis found PCOS was associated with risk of uterine cancer (odds ratio, 4.07).^40^ Furthermore, anovulation is a risk factor for uterine cancer^41^ and more common in individuals with obesity and individuals with diabetes,^42,43^ who are at greater risk of uterine cancer^44^ and thyroid cancer.^45,46^ Finally, a 2019 registry study found PCOS was significantly associated with ovarian cancer (HR, 2.16), although not thyroid cancer.^47^ Overall, underlying causes of infertility, coupled with associated obesity and diabetes, may account for either all or a substantial proportion of the observed associations between MAR treatment and risk of ovarian, uterine, and thyroid cancers.

Our findings for breast and colorectal cancer may be more robust to unmeasured confounding by the underlying cause of infertility. Recent studies found no associations between infertility history and overall risk of breast cancer^48^ or colorectal cancer,^49^ and in our study, both these cancers had the lowest observed increase in relative risk following MAR treatment. For IUI or OS, there was no association of the treatment with the risk of colorectal cancer, and for invasive breast cancer following clomiphene citrate, increased risk disappeared over time. Due to the high background rate of invasive breast cancer in the population, this association with a small increase in relative risk translated to an estimated mean of 400 extra cancers per 100 000 women treated over the course of the study period. This associated excess risk of breast cancer is similar to that following regular use of combined and progesterone-only hormonal contraceptives at ages 35 to 39 years.^50^ Findings on melanoma and its associations with fertility and pregnancy hormones (like estrogen) are mixed,^30^ so it is difficult to determine whether there is infertility-related confounding for this malignant neoplasm.

MAR treatment was associated with increased risk of hematological cancer, despite no theorized underlying biological mechanisms. This suggests other sources of bias we could not account for. International estimates show White women are more likely to undergo fertility treatments.^51^ As melanoma and hematological, uterine, breast, ovarian, and thyroid cancers are more common in White people,^52^ confounding by race and ethnicity may be responsible for the associations we observed for these malignant neoplasms.

Notably, we observed a negative association between MAR treatment and lung cancer. We posit this may reflect uncontrolled confounding by smoking behavior, with women who undergo MAR being substantially less likely to smoke before pregnancy^53^ and potentially in the prenatal period. There is evidence smoking is a risk factor for ovarian, colorectal, and breast cancers.^28^ By contrast, smoking may be protective for melanoma.^54^ Therefore, failure to control for smoking history may have attenuated our observed associations between MAR and these cancers.

### Meaning

Clinically, our findings indicate the associations of MAR with increased cancer risks may be partially or fully explained by underlying infertility conditions (ovarian, uterine, and thyroid cancer), heightened medical surveillance (breast and colorectal cancer), and demographic characteristics (melanoma), rather than MAR itself. However, given the numerous associations observed between MAR treatments and hormone-related cancers, there is a clear need for routine surveillance of patients after treatment. When advising patients considering undertaking MAR, health care professionals should emphasize cautious interpretation and communication of cancer risk.

### Unanswered Questions and Future Research

Our study highlights 2 areas for further research. MAR treatment may increase the risk of ovarian, uterine and thyroid cancers, albeit less than we observed. To improve our ability to answer this question, it is critical that we collect and make available for research information on key confounding variables at the population level. Such enhanced data collection efforts would significantly strengthen the capacity to identify and account for complex confounding pathways, ultimately improving the validity of causal inferences related to MAR and risk of cancer and other health outcomes. Second, future studies should aim to replicate the time-varying associations across cancers to test for robustness.

### Limitations

This study has some limitations. We were limited to routinely collected national data, which did not include several important confounders, such as underlying cause of infertility, race and ethnicity, individual-level socioeconomic factors (eg, education and income, which may also be markers for engagement with preventive health services), family history of cancer, smoking, and oral contraceptive use. Although the optimal emulated trial would account for all potential confounding factors, this is likely an impossible task for causal questions regarding use of MAR.^12^ Our research represents the most comprehensive attempt to address the effect of this missing confounder available, to our knowledge, through bias analysis.

Due to concerns of unmeasured confounding from cause of infertility, as well as computational feasibility, we were unable to assess a mediating effect of pregnancy. Given pregnancy may be associated with a changed risk of several hormone-related cancers^55^ it is likely there may be a different effect of MAR treatment on cancer dependent on whether a pregnancy is achieved.

We were unable to examine associations with specific medications and dosages used in ART and IUI or OS treatments. We only included women who received government reimbursement for their MAR treatments, and those who paid out-of-pocket for fertility services (such as those in same-sex relationships without a diagnosis of infertility, which is not covered under Medicare) were not included. We underascertained clomiphene citrate use between January and June 2009 and January and April 2012 due to systematic undercapture in the Pharmaceutical Benefits Scheme Dataset, which may have led to misclassification of some women as not exposed to MAR.

Additionally, our study was conducted within an Australian context, which subsidizes the use of most MAR treatments with no exceptions for age or prior children conceived, but historically did not subsidize those not deemed to be medically infertile. Thus, caution must be taken when applying the results presented here directly to other countries with different MAR eligibility criteria.

## Conclusions

In this cohort study of MAR and cancer using target trial emulation, we examined associations of 3 types of MAR treatment, ART, IUI or OS, and clomiphene citrate, with hormone-related cancers in women. Although we observed increased relative risk for most hormone-related cancers following MAR, this corresponded to only small increases in estimated absolute excess risk. Bias analysis and the inclusion of negative control cancers showed the elevated risk was potentially fully attributable to uncontrolled confounding in some cases. Clinicians and patients should be aware that a small excess risk of cancer following MAR treatment may exist, but this excess risk may be partially or fully due to the health and sociodemographic profile of women who receive MAR and increased surveillance during treatment.

## Notes

Supplement 1. eMethods. eFigure 1. Construction of eligible exposed and unexposed women, prior to partitioning to treatment and comparator cohorts eFigure 2. Hazard ratios over time for cancers with time-varying associations with MAR treatments eTable 1. Target trial specification eTable 2. Description of datasets used in linkage eTable 3. Medicare Benefits Schedule codes and Anatomic Therapeutic Chemical codes used to identify women exposed to medically assisted reproduction eTable 4. Medicare benefits schedule and Pharmaceutical Benefits Scheme item codes for identifying MAR treatment types eTable 5. *International Classification of Diseases – Oncology 3rd Edition* topographies and morphologies for cancer classification eTable 6. Numbers of incident cancers after study entry eTable 7. Deviance and likelihood ratio tests for selection of models eTable 8. Predicted number of cancers for treatment and comparator cohorts per 100,000 women eTable 9. E-values over time for time-varying hazard ratio models eTable 10. Deviance and likelihood ratio tests for selection of models eTable 11. Hazard ratios, E-values, and cumulative marginal survival difference in incident cancers (per 100,000 women) for each emulated target trial eTable 12. E-values over time for time-varying hazard ratio models

Supplement 2. Data Sharing Statement
