# Supplementary material for: Medically Assisted Reproduction and Hormone-Related Cancers
Source: JAMA Netw Open. 2026 Jul 13;9(7):e2622832. doi: 10.1001/jamanetworkopen.2026.22832 (PMC13366193; doi:10.1001/jamanetworkopen.2026.22832)
Supplement: Supplement 1. — eMethods. eFigure 1. Construction of eligible exposed and unexposed women, prior to partitioning to treatment and comparator cohorts eFigure 2. Hazard ratios over time for cancers with time-varying associations with MAR treatments eTable 1. Target trial specification eTable 2. Description of datasets used in linkage eTable 3. Medicare Benefits Schedule codes and Anatomic Therapeutic Chemical codes used to identify women exposed to medically assisted reproduction eTable 4. Medicare benefits schedule and Pharmaceutical Benefits Scheme item codes for identifying MAR treatment types eTable 5. International Classification of Diseases – Oncology 3rd Edition topographies and morphologies for cancer classification eTable 6. Numbers of incident cancers after study entry eTable 7. Deviance and likelihood ratio tests for selection of models eTable 8. Predicted number of cancers for treatment and comparator cohorts per 100,000 women eTable 9. E-values over time for time-varying hazard ratio models eTable 10. Deviance and likelihood ratio tests for selection of models eTable 11. Hazard ratios, E-values, and cumulative marginal survival difference in incident cancers (per 100,000 women) for each emulated target trial eTable 12. E-values over time for time-varying hazard ratio models [file jamanetwopen-e2622832-s001.pdf]

Supplemental Online Content

Walker AR, Venetis C, Opdal S, et al. Medically assisted reproduction and hormone-related cancers. *JAMA Netw Open*. 2026;9(7):e2622832. doi:10.1001/jamanetworkopen.2026.22832

eMethods.

eFigure 1. Construction of eligible exposed and unexposed women, prior to partitioning to treatment and comparator cohorts

eFigure 2. Hazard ratios over time for cancers with time-varying associations with MAR treatments

eTable 1. Target trial specification

eTable 2. Description of datasets used in linkage (as presented in Vajdic, Walker (13), eTable 1)

eTable 3. Medicare Benefits Schedule codes and Anatomic Therapeutic Chemical codes used to identify women exposed to medically assisted reproduction

eTable 4. Medicare benefits schedule and Pharmaceutical Benefits Scheme item codes for identifying MAR treatment types

eTable 5. International Classification of Diseases – Oncology 3rd edition topographies and morphologies for cancer classification

eTable 6. Numbers of incident cancers after study entry

eTable 7. Deviance and likelihood ratio tests for selection of models

eTable 8. Predicted number of cancers for treatment and comparator cohorts per 100,000 women

eTable 9. E-values over time for time-varying hazard ratio models

eTable 10. Deviance and likelihood ratio tests for selection of models

eTable 11. Hazard ratios, E-values, and cumulative marginal difference in incident cancers (per 100,000 women) for each emulated target trial

eTable 12. E-values over time for time-varying hazard ratio models

This supplemental material has been provided by the authors to give readers additional information about their work.

© 2026 Walker AR et al. *JAMA Network Open*. This is an open access article distributed under the terms of the CC-BY-NC-ND License, which does not permit alteration or commercial use, including those for text and data mining, AI training, and similar technologies.

eMethods

Data sources

The Australian Institute of Health and Welfare (AIHW) in conjunction with state-based Data Linkage Units conducted a bespoke linkage of jurisdictional and national population-based registries and administrative datasets (see eTable 2) to ascertain all variables. All data linkage was probabilistic. The AIHW used the Medicare Enrolment File as the linkage spine, and linked all other datasets to this spine based on individuals' given name(s) and surname, phonetic encoding keys (double metaphone) for given name(s) and surname, sex, date of birth, residential postcode, geocoded residential address, and the first 6 letters of residential address.

Target trial specification and emulation

We used the target trial emulation method to estimate the effect of exposure on cancer development, altering the exposure type and the cancer of interest (1). In brief, the method involves defining the ideal target trial for an intervention and then emulating that trial as closely as possible using observational data. Under strong assumptions, target trial emulation can estimate the true causal effect of a treatment on an outcome.(1, 2) We followed the TARGET guidelines for reporting.(3, 4) The optimal target trial is outlined in eTable 1 and shown below.

Cohort eligibility

All people recorded as female in the Australian Medicare Enrolment File (MEF) who were alive and aged between 18 and 55 years at any point between 1 January 1991 and 31 December 2018 and who had no record in the MEF of ever living in Australia's Northern Territory (due to lack of data availability) were eligible for inclusion. The MEF is a record of Australians registered to receive Medicare. Medicare is Australia's public health care insurance scheme, of which all citizens and permanent residents are eligible, with almost all Australians registered soon after birth or residency. Data on these women were linked to the Medicare Benefits Schedule dataset (MBS; subsidised health services) and Pharmaceutical Benefits Scheme dataset (PBS; subsidised medicines) to identify medical consultations, procedures and medications for fertility treatments.

Initial selection of eligible treatment and comparator women was conducted by the AIHW. The AIHW partitioned women into "Exposed" (those who had received a MAR treatment) and "Unexposed" groups (those who had not).

To form the group of MAR-exposed eligible women, the AIHW identified all women in the inclusion period who were recorded in the MBS or PBS datasets as having received a relevant benefit for any MAR treatment (See eTable 3). Though MAR services and medicines can be accessed in Australia outside of Medicare, approximately 92% of ART treatments are reimbursed through the MBS.(5, 6)

To form the group of unexposed eligible women, each MAR-exposed woman was matched with up to four women with no record of receiving MAR treatment (matched randomly with replacement across exposed MAR women). Matching occurred on birth year, residential remoteness (metropolitan or rural), parity at matched exposed woman's first MAR exposure date, and (if the matched exposed woman was parous at their first MAR) their age at their first birth +/- 365 days. All women in the unexposed group were alive on the date of the matched exposed woman's first MAR exposure date.

To match exposed and unexposed women, it was necessary to estimate their parity over time using jurisdictional Perinatal Data Collections (PDCs). As the starting date for each jurisdictional PDC data varied (eTable 4), women were only eligible for inclusion and matching once PDC data became available in their State.

Following cohort provision by the AIHW, further data cleaning steps were undertaken by researchers. eFigure 1 shows the steps taken to ascertain the cohort of eligible women for treatment strategy assignment.

Treatment strategies

Three different types of MAR treatment were identified by the presence of relevant MBS and/or PBS codes (eTable 4):

1. ART treatments (IVF or ICSI) (hereafter "ART");

© 2026 Walker AR et al. *JAMA Network Open*. This is an open access article distributed under the terms of the CC-BY-NC-ND License, which does not permit alteration or commercial use, including those for text and data mining, AI training, and similar technologies.

2. Intrauterine insemination (IUI) with ovarian stimulation with FSH *or* ART cancelled before egg retrieval (hereafter "IUI/OS"); and
3. Ovulation induction using Clomiphene Citrate (hereafter "Clomiphene Citrate").

Assignment procedure

Women were classified into treatment groups was conducted based the criteria presented in eTable 7. Assignment was based on specific MBS or PBS codes within 28 days of first MAR exposure. Overall, 93.4% of women identified as MAR-exposed by AIHW were classified into a treatment group. The remaining women did not have treatment codes relevant for the study in the allotted time and were excluded from the study. For example, this may have occurred if a woman was dispensed a MAR drug (other than Clomiphene Citrate), but there was no MBS reimbursement recorded within 28 days for initiating a MAR treatment (e.g., due to falling pregnant after filling the prescription but before starting treatment, or receiving treatment without MBS reimbursement).

A woman was assigned to the respective comparator group if she was matched to the MAR-exposed woman selected for inclusion by the AIHW. Due to an error in data extraction, a substantial proportion of comparator individuals (around 300,000) identified by the AIHW for study inclusion were not in the country at t0 and had to be excluded, as we could not determine the presence or absence of cancer. This error created an imbalance in the number of nulliparous women in the MAR treatment and comparator groups, which was accounted for later through inverse probability of treatment weighting.

As prescription data only became available from 1 July 2002, women were only assigned to the emulated trial on Clomiphene Citrate from this date.

Follow-up

Data were partitioned into 28-day windows for analysis, starting from the day of first recorded MBS or PBS code indicating MAR treatment or matched day for the comparator group. This approach was taken to approximate the length of an IVF treatment cycle, with each year approximated as 364 days (13 cycles).

Time zero (t0) was defined as the first 28-day window in which treatment assignment occurred, with follow-up beginning from the next 28-day window.

Women were followed up until either a relevant cancer outcome, 31 December 2019, or death (as recorded in the National Death Index), whichever occurred first.

Outcome definition

Hormone-related cancers were chosen as outcomes, comprising breast (invasive, in-situ), ovarian (serous, non-serous, any), uterine, melanoma (invasive, in-situ), colorectal, and thyroid cancer, identified by a record in the Australian Cancer Database (ACD). Cancers not considered hormone-related (pancreatic, lung, and haematological) were included as negative controls. Only invasive tumours were considered (International classification of disease – Oncology 3rd edition: behaviour code 3), unless specified. All cancers were classified using ICD-O3 topographies and morphologies (eTable 5).

Causal contrasts

All emulated trials used an intention-to-treat protocol.

Identifying assumptions

It was assumed that available variables appropriately controlled for residual confounding, and the likelihood of censoring did not systematically differ between those assigned to the treatment and comparator groups.

Statistical analysis

For each target trial, stabilized inverse propensity score weighting was used to control for confounding.(7) Weights were estimated using logistic regression, predicting the likelihood of assignment to the treatment group from the confounding set.

Confounding variables included:

- Age at t0;
- History of giving birth prior to t0;

© 2026 Walker AR et al. *JAMA Network Open*. This is an open access article distributed under the terms of the CC-BY-NC-ND License, which does not permit alteration or commercial use, including those for text and data mining, AI training, and similar technologies.

- Residential remoteness at t0 (metropolitan, inner regional, remote/very remote);
- Average area-based Index of Relative Socioeconomic Disadvantage percentile, based on available lifetime data for Australian area of residence;
- Subsidised diabetes medication record prior to t0; and
- Cancer registry notified cancer diagnosis prior to t0.

Confounding variables included in our study were determined based on the directed acyclic graph in Walker et al.(8) and the available data.

Following weighting, four nested flexible parametric survival models (9) with robust variance estimators were run. To account for similarities between treated women and their matched comparators, we used cluster-robust variance estimators, treating each MAR-treated woman and her matched comparators as a cluster.

We had originally planned to use Cox regression in our analysis. However, there were clear benefits to switching to flexible parametric survival models. Flexible parametric survival models estimate a parametric model of the baseline hazard, which allows for prediction of survival over time. These models also allow underlying hazards to vary for specified categorical variables, which allows the hazard ratio to vary over time. When no variables are specified as having time-varying hazards, flexible parametric models closely approximate Cox regression. Our model of the baseline hazard included two splines. We also included two splines for the hazard function of age at first treatment based on preliminary modelling suggesting that this would provide a better model fit. We tested four models to ascertain the number of splines needed in the hazard function of treatment to determine the best fitting model. The models tested include a model with no time-varying effect of treatment, and three models with time varying effects of treatment, altering the number of splines in the hazard function (0, 1, or 3 splines, equally spaced). Flexible parametric models were calculated using the stpm3 command in STATA.

The model selection steps included:

Comparing each time-varying model to the non-time-varying model. If one model outperformed the non-time-varying model, we selected that model. If multiple models outperformed the non-time-varying model, we went to step 2. Otherwise, we selected the non-time-varying model;

Comparing the remaining models to the simplest model that outperformed the non-time varying model. If one model outperformed the simplest model, we selected that model. If multiple models outperformed the simplest model, we repeated step 2. Otherwise, we selected the simplest model.

For emulated trials where the non-time-varying model was preferred, the mean hazard ratio across the full follow-up period is reported. For emulated trials where a time-varying effect of treatment was preferred, the hazard ratio changes over time are shown graphically.

For each model, the cumulative marginal difference in incident cancers between the treatment and comparator groups over time was estimated and reported as the number of additional cancer cases expected per 100,000 women at year 1, and then every five years starting at year 5. This was done using the *standsurv* postestimation command in STATA.(10) Briefly, the command models the average survival curves for the entire population assuming either treatment or non-treatment, controlling for the included confounding variables. By comparing the marginal survival curve assuming treatment to the marginal survival curve assuming non-treatment at different times from t0, one can determine how many extra incident cancers were predicted after a certain number of years. This process is similar to comparing Kaplan-Meier survival curves, but crucially provides an estimate of the survival difference adjusted for available confounding variables.

In each emulated trial, E-values(11) were calculated to assess the likelihood uncontrolled confounding impacted our findings. Briefly, E-values are measured on the risk-ratio scale and reflect the weakest association between an uncontrolled confounder and both the exposure and outcome, that could fully explain the observed effect. A full discussion of E-values and their use in studies of MAR treatments and health outcomes is given in Walker, Venetis (8). When hazard ratios changed over time, the E-value was assessed at year 1, and then every five years starting at year 5.

We conducted a complete case analysis of the data. We assumed data were missing at random, that is, that missingness in the outcome (due to either lack of detection during the study period or

© 2026 Walker AR et al. *JAMA Network Open*. This is an open access article distributed under the terms of the CC-BY-NC-ND License, which does not permit alteration or commercial use, including those for text and data mining, AI training, and similar technologies.

migration out of the country) were dependent on our observed variables (particularly age, remoteness, and socioeconomic status).

Analysis was conducted using SAS version 9 and STATA 18.

Study registration

This study, including rationale, target trial specification, and analysis plan, was pre-registered on the Open Science Framework at https://osf.io/rk9n6.(12) Although the study was pre-registered, some elements of the design and analysis were adapted during implementation to strengthen feasibility and validity.

Ethics approval

The study was approved by all relevant human research ethics committees (HRECs) including the AIHW HREC (EO2019/5/1061). Data was accessed and used under a waiver of informed consent. Researchers were granted access to linked anonymised data.

Role of the funding Source

This project was funded by the National Health and Medical Research Council (NHMRC: APP1164852). The funders had no role in the design of the study, the collection, analysis or interpretation of the data, the writing of the manuscript or the decision to submit the manuscript for publication.

Changes from registered protocol

We note that, compared to our registered protocol, we opted to use flexible parametric survival models over Cox regression to allow the recovery of cumulative marginal differences, and a changing proportional hazard over time. We were unable to assess the effect of gonadotrophins or anti-gonadotropin-releasing hormone use due to issues with completeness of data capture for these medicines. We chose not to assess dose effect of treatment due to the computational feasibility of conducting a g-methods-based analysis with large data across a long period of time for multiple models, and the impact of unmeasured confounding from cause of infertility (which would likely be exacerbated in the calculation of a dose effect). People with cancers of the same topography as the target cancer before t0 were not excluded, as our cancer definitions included multiple histologies within the same topography that could be considered primary cancers. Sensitivity analyses on limiting the time of follow-up to ascertain changes in the hazard ratio were not run due to flexible parametric survival models accounting for the changing hazard ratios over time. The sensitivity analysis excluding those indicated as having poor linkage to children's birth records was not run as a linkage quality indicator was not provided to researchers, though researchers removed women who had a birth recorded after their death was recorded prior to analysis.

© 2026 Walker AR et al. *JAMA Network Open*. This is an open access article distributed under the terms of the CC-BY-NC-ND License, which does not permit alteration or commercial use, including those for text and data mining, AI training, and similar technologies.

References

1. Hernán MA, Robins JM. Using big data to emulate a target trial when a randomized trial is not available. American Journal of Epidemiology. 2016;183(8):758-64.
2. Hernán MA, Robins JM. Causal Inference: What If. Boca Raton: Chapman & Hall/CRC; 2020.
3. Hansford HJ, Cashin AG, Jones MD, Swanson SA, Islam N, Dahabreh IJ, et al. Development of the TrAnsparent ReportinG of observational studies Emulating a Target trial (TARGET) guideline. British Medical Journal Open. 2023;13(9):e074626.
4. Cashin AG, Hansford HJ, Hernán MA, Swanson SA, Lee H, Jones MD, et al. Transparent reporting of observational studies emulating a target trial: the TARGET Statement. British Medical Journal. 2025;390.
5. Australian Government Services Australia. Medicare Item Reports http://medicarestatistics.humanservices.gov.au/statistics/mbs_item.jsp2025 [updated 26 Feb 2025. Available from: http://medicarestatistics.humanservices.gov.au/statistics/mbs_item.jsp.
6. Newman JE, Kotevski DP, Paul RC, Chambers GM. Assisted reproductive technology in Australia and New Zealand 2022. Sydney: National Perinatal Epidemiology and Statistics Unit, the University of New South Wales, Sydney. 2024:1-91.
7. Robins JM, Hernan MA, Brumback B. Marginal structural models and causal inference in epidemiology. Epidemiology. 2000;11(5):550-60.
8. Walker AR, Venetis CA, Opdahl S, Chambers GM, Jorm LR, Vajdic CM. Estimating the impact of bias in causal epidemiological studies: the case of health outcomes following assisted reproduction. Human Reproduction. 2024;39(5):869-75.
9. Royston P, Lambert PC. Flexible parametric survival analysis using Stata: Beyond the Cox model: Stata press College Station, TX; 2011.
10. Lambert P. STANDSURV: Stata module to compute standardized (marginal) survival and related functions. Statistical Software Components 2021;S458991.
11. VanderWeele TJ, Ding P. Sensitivity analysis in observational research: introducing the E-value. Annals of Internal Medicine. 2017;167(4):268-74.
12. Walker A, Vajdic C, Chambers G, Venetis C, Opdahl S. Cancer Risk aftEr medicAlly assisTed rEproduction (The CREATE Project). 2023.
13. Vajdic CM, Walker AR, Anazodo AC, Hacker NF, Chapman M, Opdahl S, et al. Cancer Incidence in Women After Medically Assisted Reproduction. JAMA Network Open. 2026;9(3):e261332-e.
14. Australian National Audit Office. Integrity of Medicine Enrolment Data 2024 [Available from: https://www.anao.gov.au/work/performance-audit/integrity-medicare-enrolment-data.
15. Australian Institute of Health and Welfare. Medicare Benefits Schedule data collection 2024 [Available from: https://www.aihw.gov.au/about-our-data/our-data-collections/medicare-benefits-schedule-mbs.
16. Australian Institute of Health and Welfare. Pharmaceutical Benefits Scheme data collection 2024 [Available from: https://www.aihw.gov.au/about-our-data/our-data-collections/pharmaceutical-benefits-scheme.
17. Australian Institute of Health and Welfare. National Death Index 2024 [Available from: https://www.aihw.gov.au/about-our-data/our-data-collections/national-death-index.
18. Australian Institute of Health and Welfare. Australian Cancer Database 2024 [Available from: https://www.aihw.gov.au/about-our-data/our-data-collections/australian-cancer-database.

© 2026 Walker AR et al. *JAMA Network Open*. This is an open access article distributed under the terms of the CC-BY-NC-ND License, which does not permit alteration or commercial use, including those for text and data mining, AI training, and similar technologies.

eFigures

eFigure 1: Construction of eligible exposed and unexposed women, prior to partitioning to treatment and comparator cohorts

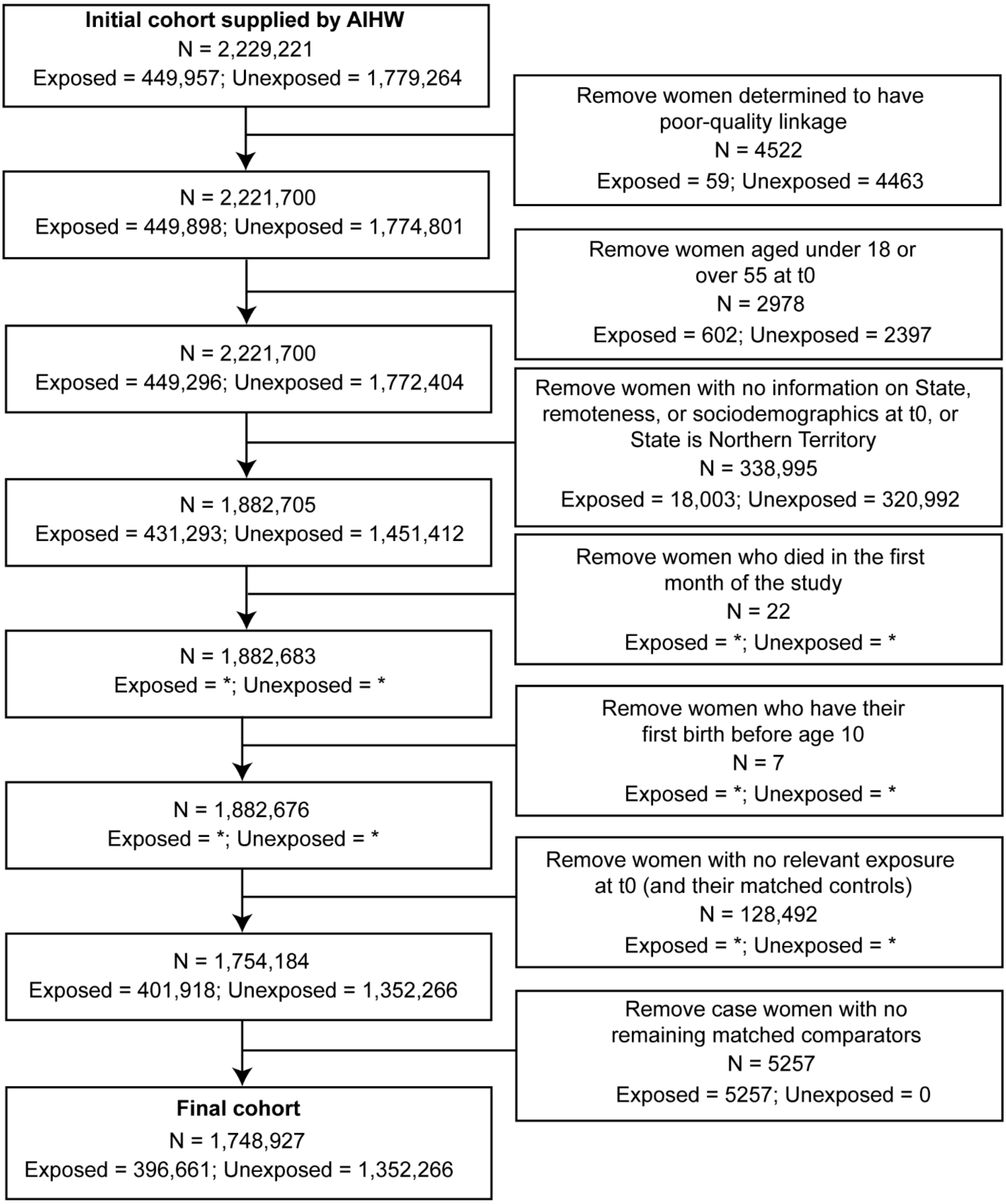

*Censored to prevent re-identification from small cell sizes
AIHW: Australian Institute of Health and Welfare; t0: Time 0

© 2026 Walker AR et al. *JAMA Network Open*. This is an open access article distributed under the terms of the CC-BY-NC-ND License, which does not permit alteration or commercial use, including those for text and data mining, AI training, and similar technologies.

eFigure 2: Hazard ratios over time for cancers with time-varying associations with MAR treatments (No prior cancer sensitivity analysis). Figures start from 6 months to improve figure readability.

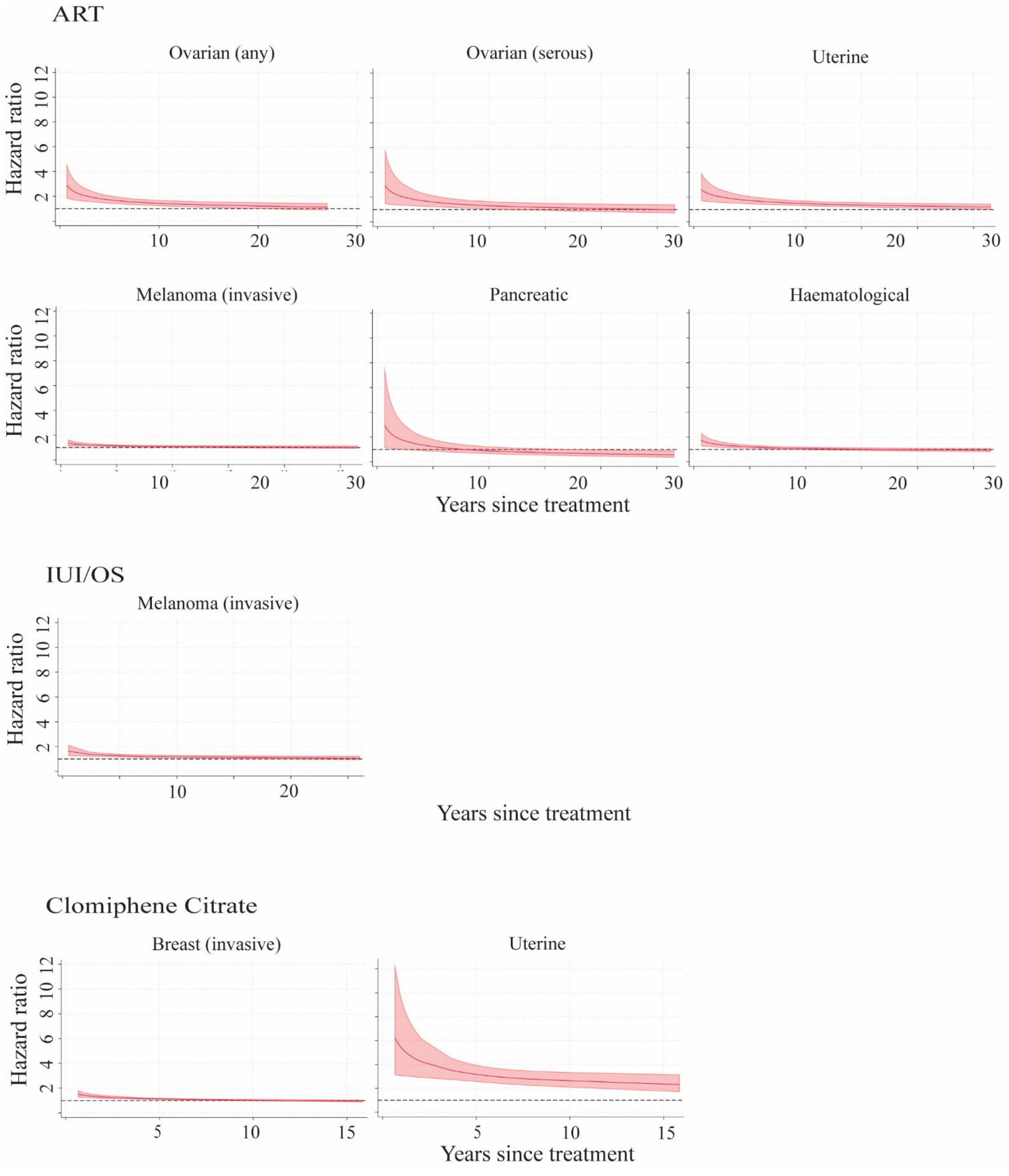

© 2026 Walker AR et al. *JAMA Network Open*. This is an open access article distributed under the terms of the CC-BY-NC-ND License, which does not permit alteration or commercial use, including those for text and data mining, AI training, and similar technologies.

eTables

eTable 1: Target trial specification.

	Target trial specification
Eligibility criteria	Australian women aged between 18 and 55 between January 1991 and 31 December 2018 who were eligible to receive a MAR treatment and had not previously received a MAR treatment.
Treatment strategies	Treatment group: undergoing a MAR treatment (ART, IUI/OS) or receiving a Clomiphene prescription, depending on the type of treatment under examination; Comparator group: not undergoing a MAR treatment of any type.
Assignment procedures	Participants are randomly assigned to the treatment group and comparator group (four comparator participants assigned for every treatment participant).
Follow-up	Until end of study period or death.
Outcomes	Diagnosis of the target cancer (invasive breast, in-situ breast, any ovarian, serous ovarian, non-serous ovarian, uterine, thyroid, colorectal, invasive melanoma, in-situ melanoma).
Causal contrasts	Per-protocol
Identifying assumptions	Randomisation occurred during group assignment, and censoring events during follow-up do not occur systematically based on the presence or absence of the exposure.
Data analysis plan	Appropriate survival analysis that estimates both the hazard ratio and survival difference of those assigned to the treatment group compared to the comparator group.

MAR=Medically Assisted Reproduction; ART=Assisted Reproductive Technology; IUI/OS=Intrauterine Insemination/Ovarian Stimulation

© 2026 Walker AR et al. *JAMA Network Open*. This is an open access article distributed under the terms of the CC-BY-NC-ND License, which does not permit alteration or commercial use, including those for text and data mining, AI training, and similar technologies.

© 2026 Walker AR et al. *JAMA Network Open*. This is an open access article distributed under the terms of the CC-BY-NC-ND License, which does not permit alteration or commercial use, including those for text and data mining, AI training, and similar technologies.

eTable 2: Description of datasets used in linkage (as presented in Vajdic, Walker (13), eTable 1).

Dataset	Timeframe	Original purpose and setting	Use in cohort
**National datasets**			
Medicare Enrolment File (MEF)(14)	1 Jan 1991 - 31 Dec 2019	Administrative data collection of all individuals enrolled in Medicare, Australia's national public health insurance program, including names and addresses. Almost all Australians are enrolled soon after birth; immigrants are enrolled when they become eligible.	Used to identify women eligible for cohort entry and derive information related to residential geographic location (state, residential remoteness, and socioeconomic status).
Medicare Benefits Schedule (MBS)(15)	1 Jan 1991 - 31 Dec 2019	Administrative data collection of all health service claims through the Medicare Benefits Schedule. Data provided by healthcare providers who claim from the scheme.	Used to identify women who received a MAR treatment in the study period. Only MAR-relevant items provided to researchers (See eTable 11)
Pharmaceutical Benefits Scheme (PBS)(16)	1 Jul 2002 - 31 Dec 2019	Administrative data collection of all drugs dispensed through the Pharmaceutical Benefits Scheme, Australia's national public drug reimbursement program. Data provided by pharmacists who claim from the scheme.	Used to identify women who were dispensed a MAR-relevant drug in the study period, and the type of drug. Also used to identify whether the woman was dispensed drugs pertaining to diabetes (Anatomical Therapeutic Chemical code A10) (See eTable 11).
National Death Index (NDI)(17)	1 Jan 1991 - 31 Dec 2019	Mandated registry of all deaths occurring in Australia. Obtained from the Australian Institute of Health and Welfare.	Used to identify when people died to censor person-time at risk in analysis.
Australian Cancer Database (ACD)(18)	1 Jan 1982 - 31 Dec 2019	Collection of all notifiable cancers notified to the Australian population-based cancer registries.	Used to identify when cancer was diagnosed, as well as the type of cancer (topography and morphology).
State datasets			
Perinatal Data Collections (PDCs) for NSW, ACT, QLD, VIC, WA*, and TAS	**NSW:** 1 Jan 1994 – 30 Jun 2019<br>**ACT:** 1 Jan 1997 – 1 Dec 2017<br><br>**QLD:** 1 Jan 2007 - 31 Dec 2019<br>**VIC:** 1 Jan 1999 - 20 Oct 2022	Administrative data collections of all pregnancies, generally beyond 20 weeks gestation. Victoria also includes pregnancies above 400 grams in weight at birth if not over 20 weeks. State collections differ, but include date of birth, status of the birth (live or still), plurality, parity, complications of pregnancy, and other relevant data items.	Used to determine mother's history of pregnancy, including previous children and parity of time at study entry, and pregnancy characteristics.

© 2026 Walker AR et al. *JAMA Network Open*. This is an open access article distributed under the terms of the CC-BY-NC-ND License, which does not permit alteration or commercial use, including those for text and data mining, AI training, and similar technologies.

	**SA:** 1 Jan 1991 - 31 Dec 2019<br>**WA:** 1 Jan 1991 - 31 Dec 2020<br>**TAS:** 1 Jan 2005 - 31 Dec 2019		
Registry of Births, Deaths, and Marriages (RBDM) for NSW, ACT, QLD, VIC, WA, and TAS	**NSW:** 1 Jan 1994 – 30 Jun 2019<br>**ACT:** 1 Jan 1997 – 1 Dec 2017<br>**QLD:** 1 Jan 2007 - 31 Dec 2018<br>**VIC:** 1 Jan 1999 - 20 Oct 2022<br>**SA:** 1 Jan 1991 - 31 Dec 2019<br>**WA:** 1 Jan 1991 - 31 Dec 2020<br>**TAS:** 1 Jan 2005 - 31 Dec 2019	Mandated registers of all births recorded in each State and Territory, including information on mothers and fathers.	Used in combination with the perinatal data collections to determine details of mother's history of pregnancy and parity.

NSW=New South Wales; ACT=Australian Capital Territory; QLD=Queensland; VIC=Victoria; SA=South Australia; WA=Western Australia; TAS=Tasmania.
*Known as the "Western Australia Midwife Notification System".

© 2026 Walker AR et al. *JAMA Network Open*. This is an open access article distributed under the terms of the CC-BY-NC-ND License, which does not permit alteration or commercial use, including those for text and data mining, AI training, and similar technologies.

eTable 3: Medicare Benefits Schedule (MBS) codes and Anatomic Therapeutic Chemical (ATC) codes used to identify women exposed to medically assisted reproduction.

Dataset	Code	Description
Medicare Benefits Schedule	9065	ASSISTED REPRODUCTIVE TECHNOLOGIES involving handling of both human ova and sperm including invitro fertilisation or gamete intra-fallopian transfer or similar techniques, when rendered in conjunction with item 4194 (Laparoscopy) or Item 3004(82) (Ova flushing), all such services rendered in one treatment cycle which commenced on or after 1 August 1989
	13200	ASSISTED REPRODUCTIVE TECHNOLOGIES SUPEROVULATED TREATMENT CYCLE PROCEEDING TO OOCYTE RETRIEVAL, involving the use of drugs to induce superovulation, and including quantitative estimation of hormones, semen preparation, ultrasound examinations, all treatment counselling and embryology laboratory services but excluding artificial insemination or transfer of frozen embryos or donated embryos or ova or a service to which item13201, 13202, 13203, 13218 applies - being services rendered during 1 treatment cycle - INITIAL cycle in a single calendar year
	13201	ASSISTED REPRODUCTIVE TECHNOLOGIES SUPEROVULATED TREATMENT CYCLE PROCEEDING TO OOCYTE RETRIEVAL, involving the use of drugs to induce superovulation, and including quantitative estimation of hormones, semen preparation, ultrasound examinations, all treatment counselling and embryology laboratory services but excluding artificial insemination or transfer of frozen embryos or donated embryos or ova or a service to which item13200, 13202, 13203, 13218 applies - being services rendered during 1 treatment cycle - each cycle SUBSEQUENT to the first in a single calendar year
	13202	ASSISTED REPRODUCTIVE TECHNOLOGIES SUPEROVULATED TREATMENT CYCLE THAT IS CANCELLED BEFORE OOCYTE RETRIEVAL, involving the use of drugs to induce superovulation and including quantitative estimation of hormones, semen preparation, ultrasound examinations, but excluding artificial insemination or transfer of frozen embryos or donated embryos or ova or a service to which Item 13200, 13201, 13203, 13218, applies being services rendered during 1 treatment cycle
	13203	Ovulation monitoring services for artificial insemination or gonadotrophin, stimulated ovulation induction, including quantitative estimation of hormones and ultrasound examinations, being services rendered during one treatment cycle but excluding a service to which item 13200, 13201, 13202, 13212, 13215 or 13218 applies
	13206	ASSISTED REPRODUCTIVE TECHNOLOGIES TREATMENT CYCLE using either the natural cycle or oral medication only to induce oocyte growth and development, and including quantitative estimation of hormones, semen preparation, ultrasound examinations, all treatment counselling and embryology laboratory services but excluding artificial insemination, frozen embryo transfer or donated embryos or ova or treatment involving the use of injectable drugs to induce superovulation being services rendered during 1 treatment cycle but only if rendered in conjunction with a service to which item 13212 applies
	13209	PLANNING and MANAGEMENT of a referred patient by a specialist for the purpose of treatment by assisted reproductive technologies or for artificial insemination payable once only during 1 treatment cycle
	13212	Oocyte retrieval for the purpose of assisted reproductive technologies—only if rendered in connection with a service to which item 13200 or 13201 applies (H)
	13215	Transfer of embryos or both ova and sperm to the uterus or fallopian tubes, excluding artificial insemination-only if rendered in connection with a service to which item 13200, 13201 or 13218 applies, being services rendered in one treatment cycle
	13218	PREPARATION of frozen or donated embryos or donated oocytes for transfer to the uterus or fallopian tubes, by any means and including quantitative estimation of hormones and all treatment counselling but excluding artificial insemination services rendered in 1 treatment cycle and excluding a service to which item 13200, 13201, 13202, 13203, 13212 applies

© 2026 Walker AR et al. *JAMA Network Open*. This is an open access article distributed under the terms of the CC-BY-NC-ND License, which does not permit alteration or commercial use, including those for text and data mining, AI training, and similar technologies.

	13221	Preparation of semen for the purpose of artificial insemination-only if rendered in connection with a service to which item 13203 applies
	13251	INTRACYTOPLASMIC SPERM INJECTION for the purposes of assisted reproductive technologies, for male factor infertility, excluding a service to which Item 13203 or 13218 applies
Pharmaceutical Benefits Scheme (identified from Anatomic Therapeutic Chemical codes)	G03GB02	Clomifene
	G03GA01	Chorionic gonadotrophin
	G03GA02	Human menopausal gonadotrophin
	G03GA04	Urofollitropin
	G03GA05	Follitropin alfa
	G03GA06	Follitropin beta
	G03GA07	Lutropin alfa
	G03A08	Choriogonadotrophin alfa
	G03GA09	Corifollitropin alfa
	G03GA10	Follitropin delta
	H01CC01	Ganirelix
	H01CC02	Cetrorelix

© 2026 Walker AR et al. *JAMA Network Open*. This is an open access article distributed under the terms of the CC-BY-NC-ND License, which does not permit alteration or commercial use, including those for text and data mining, AI training, and similar technologies.

eTable 4: Medicare Benefits Schedule (MBS) and Pharmaceutical Benefits Scheme (PBS) item codes for identifying MAR treatment types (as presented in Vajdic, Walker (13), eTable 2).

	MBS item codes[a, b]	PBS item codes
**Before Jan 2010**		
ART (IVF + ICSI)	13200 OR 13206 OR 13212	
IUI/OS	13203	
Clomiphene Citrate[c]		01211R (from 1 July 2002, excluding 1 Jan 2009 to 30 Jun 2009)
**After Jan 2010**		
ART (IVF + ICSI)	13200 OR 13201 OR 13206 OR 13212	
IUI/OS	13202 OR 13203	
Clomiphene Citrate[c]		01211R (from 1 July 2002, excluding 1 Jan 2012 to 30 Apr 2012)

[a]Presence or one or more of these codes within a 30-day window in the relevant dataset indicated the receipt of the relevant treatment in that window
[b]Using publicly available data on ART cycles delivered in Australia in 2022 and the number of relevant Medicare claims in the same year, we estimate approximately 92% of ART cycles delivered in Australia are captured in Medicare records. (5, 6)
[c]Our analysis of reproductive medicines was restricted to those not classified as Section 100 fertility drugs by the Pharmaceutical Benefits Scheme, as the recording of dispensations for these drugs was handled by Services Australia for most of the study period and not present in the supplied data. Prior to collection of medicines under the copayment threshold in May 2012, Clomiphene Citrate was under the MBS copayment threshold from 1 Jan 2009 to 30 Jun 2009 and 1 Jan 2012 to 30 Apr 2012, leading to no ascertainment during these periods.
MBS=Medicare Benefits Schedule; PBS=Pharmaceutical Benefits Scheme; MAR=Medically assisted reproduction; ART=Assisted reproduction technology; IVF=In-vitro fertilisation; IUI/OS=Intrauterine Insemination/Ovarian Stimulation

© 2026 Walker AR et al. *JAMA Network Open*. This is an open access article distributed under the terms of the CC-BY-NC-ND License, which does not permit alteration or commercial use, including those for text and data mining, AI training, and similar technologies.

eTable 5: International classification of Diseases – Oncology 3rd edition topographies and morphologies for cancer classification.

Target cancer	ICD-O-3 topography	ICD-O-3.1 morphology (all behaviour code 3 unless otherwise stated)
Breast (invasive)	C50	All
Breast (in-situ)	C50	All (behaviour code 2)
Ovarian (all)	C56	All
Ovarian (serous)	C56	8441
Ovarian (non-serous)	C56	All except 8441
Uterine	C54, C55	All
Melanoma (invasive)	C44	872-879
Melanoma (in-situ)	C44	872-879 (behaviour code 2)
Colorectal (excluding anus)	C18-C20	All
Thyroid	C73	All
Pancreatic	C25	All
Lung	C33-C34	All
Haematological	C00-C80	959-998

© 2026 Walker AR et al. *JAMA Network Open*. This is an open access article distributed under the terms of the CC-BY-NC-ND License, which does not permit alteration or commercial use, including those for text and data mining, AI training, and similar technologies.

eTable 6: Numbers of incident cancers after study entry. Percentages are relative to the size of the respective treatment/comparator cohort.

Cancer type	ART		IUI/OS		Clomiphene Citrate	
	Treatment	Comparator	Treatment	Comparator	Treatment	Comparator
Breast (invasive)	2,588 (1.5%)	7,541 (1.3%)	1,445 (1.9%)	4,245 (1.6%)	1,191 (0.8%)	3,847 (0.7%)
Breast (in-situ)	212 (0.2%)	567 (0.1%)	79 (0.2%)	230 (0.1%)	170 (0.1%)	529 (0.1%)
Ovarian	221 (0.1%)	461 (0.1%)	124 (0.2%)	266 (0.1%)	82 (0.1%)	198 (0.0%)
Ovarian (serous)	103 (0.1%)	237 (0.0%)	60 (0.1%)	127 (0.0%)	32 (0.0%)	81 (0.0%)
Ovarian (non-serous)	118 (0.1%)	225 (0.0%)	64 (0.1%)	139 (0.1%)	50 (0.0%)	117 (0.0%)
Uterine	297 (0.2%)	632 (0.1%)	180 (0.2%)	364 (0.1%)	193 (0.1%)	192 (0.0%)
Thyroid	466 (0.3%)	1,295 (0.2%)	232 (0.3%)	695 (0.3%)	362 (0.2%)	977 (0.2%)
Colorectal (excluding anus)	447 (0.3%)	1,288 (0.2%)	234 (0.3%)	758 (0.3%)	228 (0.1%)	661 (0.1%)
Melanoma (invasive)	871 (0.5%)	2,546 (0.4%)	538 (0.7%)	1,437 (0.6%)	565 (0.4%)	1,618 (0.3%)
Melanoma (in-situ)	485 (0.4%)	1,490 (0.3%)	217 (0.5%)	530 (0.3%)	580 (0.4%)	1,592 (0.3%)
Pancreatic	51 (0.0%)	186 (0.0%)	32 (0.0%)	120 (0.0%)	20 (0.0%)	69 (0.0%)
Lung	173 (0.1%)	684 (0.1%)	90 (0.1%)	401 (0.2%)	64 (0.0%)	231 (0.0%)
Haematological	465 (0.3%)	1,297 (0.2%)	286 (0.4%)	716 (0.3%)	257 (0.2%)	739 (0.1%)

ART=Assisted Reproductive Technology; IUI/OS=Intrauterine Insemination/Ovarian Stimulation

© 2026 Walker AR et al. *JAMA Network Open*. This is an open access article distributed under the terms of the CC-BY-NC-ND License, which does not permit alteration or commercial use, including those for text and data mining, AI training, and similar technologies.

eTable 7: Deviance and likelihood ratio tests for selection of models.

MAR treatment type	Deviance difference from no time-varying			Likelihood ratio test to non time varying			Deviance difference from 0 spline		Likelihood ratio test to 0 spline		Deviance difference from 1 spline	Likelihood ratio test to 1 spline	
Cancer type	0 splines	1 spline	3 splines	0 splines	1 spline	3 splines	1 spline	3 splines	1 spline	3 splines	3 splines	3 splines	Final model
**ART**													
Breast (invasive)	1.41	1.53	2.44	0.236	0.465	0.656	0.13	1.03	0.722	0.794	0.90	0.636	Non-time varying
Breast (in-situ)	0.74	0.77	1.31	0.389	0.682	0.860	0.02	0.57	0.879	0.904	0.54	0.762	Non-time varying
Ovarian	8.57	10.26	12.79	0.003	0.006	0.012	1.69	4.22	0.193	0.239	2.53	0.283	0 spline
Ovarian (serous)	5.36	7.59	9.73	0.021	0.023	0.045	2.22	4.36	0.136	0.225	2.14	0.343	0 spline
Ovarian (non-serous)	3.12	3.24	5.76	0.077	0.198	0.218	0.12	2.64	0.734	0.451	2.52	0.283	Non-time varying
Uterine	7.92	9.84	10.69	0.005	0.007	0.030	1.92	2.76	0.166	0.430	0.84	0.656	0 spline
Thyroid	0.00	0.01	0.82	0.949	0.997	0.936	0.00	0.81	0.965	0.847	0.81	0.667	Non-time varying
Colorectal	2.82	2.93	3.42	0.093	0.232	0.490	0.10	0.60	0.750	0.897	0.50	0.780	Non-time varying
Melanoma (invasive)	3.64	4.76	11.43	0.057	0.092	0.022	1.12	7.80	0.289	0.050	6.67	0.036	3 spline
Melanoma (in-situ)	0.13	1.91	2.07	0.719	0.385	0.723	1.78	1.94	0.182	0.585	0.16	0.923	Non-time varying
Pancreatic	6.95	6.98	8.71	0.008	0.030	0.069	0.03	1.76	0.855	0.624	1.73	0.422	0 spline
Lung	0.07	0.79	2.39	0.797	0.673	0.664	0.73	2.32	0.394	0.508	1.60	0.450	Non-time varying
Haematological	9.84	10.23	10.34	0.002	0.006	0.035	0.38	0.50	0.535	0.920	0.11	0.945	0 spline
**IUI/OS**													
Breast (invasive)	1.29	1.96	5.07	0.257	0.375	0.281	0.67	3.78	0.412	0.286	3.10	0.212	Non-time varying
Breast (in-situ)	1.30	2.50	4.72	0.253	0.286	0.317	1.20	3.41	0.273	0.332	2.21	0.331	Non-time varying
Ovarian	0.01	0.45	0.85	0.910	0.797	0.932	0.44	0.84	0.507	0.840	0.40	0.820	Non-time varying
Ovarian (serous)	0.46	1.42	NA	0.499	0.492	NA	0.96	NA	0.327	NA	NA	NA	Non-time varying
Ovarian (non-serous)	0.45	0.45	9.90	0.503	0.797	0.042	0.00	9.45	0.948	0.024	9.44	0.009	3 spline
Uterine	0.54	0.74	3.63	0.462	0.691	0.458	0.20	3.09	0.656	0.378	2.89	0.235	Non-time varying
Thyroid	0.00	0.01	5.76	0.953	0.997	0.218	0.00	5.75	0.964	0.124	5.75	0.056	Non-time varying
Colorectal	2.13	2.23	2.74	0.144	0.327	0.602	0.10	0.61	0.750	0.895	0.51	0.776	Non-time varying
Melanoma (invasive)	4.58	5.61	9.42	0.032	0.061	0.051	1.02	4.84	0.312	0.184	3.82	0.148	0 spline
Melanoma (in-situ)	0.73	0.88	2.59	0.393	0.645	0.628	0.15	1.86	0.699	0.601	1.71	0.424	Non-time varying
Pancreatic	0.44	2.55	NA	0.507	0.280	NA	2.11	NA	0.147	NA	NA	NA	Non-time varying
Lung	0.00	0.27	0.71	0.960	0.872	0.950	0.27	0.71	0.602	0.871	0.44	0.803	Non-time varying

© 2026 Walker AR et al. *JAMA Network Open*. This is an open access article distributed under the terms of the CC-BY-NC-ND License, which does not permit alteration or commercial use, including those for text and data mining, AI training, and similar technologies.

Haematological	1.16	1.35	8.26	0.282	0.509	0.082	0.20	7.11	0.658	0.069	6.91	0.032	Non-time varying
**Clomiphene Citrate**													
Breast (invasive)	13.28	17.57	18.98	0.000	0.000	0.001	4.30	5.71	0.038	0.127	1.41	0.494	1 spline
Breast (in-situ)	0.49	0.61	0.92	0.484	0.737	0.921	0.12	0.43	0.729	0.933	0.31	0.854	Non-time varying
Ovarian	2.05	2.32	5.38	0.152	0.313	0.250	0.28	3.33	0.600	0.343	3.06	0.217	Non-time varying
Ovarian (serous)	0.02	3.82	6.40	0.886	0.148	0.171	3.80	6.38	0.051	0.094	2.59	0.274	Non-time varying
Ovarian (non-serous)	2.40	2.94	6.65	0.121	0.230	0.155	0.53	4.25	0.465	0.236	3.72	0.156	Non-time varying
Uterine	5.60	6.02	10.56	0.018	0.049	0.032	0.42	4.96	0.518	0.175	4.54	0.103	0 spline
Thyroid	0.74	0.84	1.62	0.389	0.659	0.806	0.09	0.87	0.761	0.832	0.78	0.677	Non-time varying
Colorectal	1.64	4.09	5.63	0.200	0.129	0.229	2.45	3.99	0.117	0.263	1.54	0.464	Non-time varying
Melanoma (invasive)	0.12	0.12	5.71	0.725	0.940	0.222	0.00	5.59	0.985	0.134	5.59	0.061	Non-time varying
Melanoma (in-situ)	0.74	4.53	5.38	0.390	0.104	0.251	3.79	4.64	0.052	0.200	0.85	0.653	Non-time varying
Pancreatic	0.21	0.21	1.96	0.649	0.901	0.742	0.00	1.76	0.971	0.625	1.75	0.416	Non-time varying
Lung	0.41	0.43	1.41	0.523	0.808	0.843	0.02	1.00	0.890	0.802	0.98	0.613	Non-time varying
Haematological	0.66	3.24	7.66	0.418	0.198	0.105	2.59	7.00	0.108	0.072	4.42	0.110	Non-time varying

MAR = Medically Assisted Reproduction; ART=Assisted Reproductive Technology; IUI/OS=Intrauterine Insemination/Ovarian Stimulation. NA indicates model did not fit

© 2026 Walker AR et al. *JAMA Network Open*. This is an open access article distributed under the terms of the CC-BY-NC-ND License, which does not permit alteration or commercial use, including those for text and data mining, AI training, and similar technologies.

eTable 8: Predicted number of cancers for treatment and comparator cohorts per 100,000 women.

	Cancer type	1 year	5 years	10 years	15 years	20 years	25 years
**MAR treatment**	**ART**						
	Breast (invasive)	35 (31, 38)	318 (301, 335)	979 (937, 1021)	1953 (1874, 2033)	3182 (3058, 3306)	4631 (4443, 4818)
	Breast (in-situ)	5 (4, 7)	51 (42, 60)	187 (160, 215)	432 (371, 494)	790 (653, 927)	1261 (993, 1528)
	Ovarian	7 (4, 9)	36 (28, 44)	86 (72, 100)	155 (132, 177)	252 (217, 286)	376 (319, 433)
	Ovarian (serous)	3 (1, 4)	15 (10, 20)	38 (29, 47)	71 (56, 86)	120 (96, 144)	186 (145, 227)
	Ovarian (non-serous)	3 (1, 4)	18 (13, 23)	45 (35, 54)	83 (67, 99)	136 (111, 161)	203 (164, 242)
	Uterine	6 (4, 8)	32 (25, 39)	88 (75, 102)	182 (159, 206)	350 (308, 391)	627 (547, 706)
	Thyroid	14 (11, 16)	93 (82, 103)	209 (189, 230)	350 (317, 383)	515 (467, 563)	700 (630, 769)
	Colorectal	8 (6, 9)	58 (50, 65)	157 (140, 173)	308 (277, 339)	541 (489, 592)	864 (778, 949)
	Melanoma (invasive)	29 (22, 35)	185 (165, 206)	400 (365, 434)	631 (582, 681)	962 (893, 1032)	1388 (1270, 1506)
	Melanoma (in-situ)	30 (25, 35)	197 (177, 216)	534 (484, 583)	1117 (1006, 1228)	1910 (1682, 2138)	2889 (2484, 3294)
	Pancreatic	2 (0, 3)	8 (4, 12)	19 (13, 26)	36 (26, 47)	61 (44, 79)	95 (65, 124)
	Lung	1 (1, 2)	13 (10, 16)	45 (37, 53)	105 (88, 122)	217 (184, 250)	416 (351, 481)
	Haematological	13 (9, 17)	73 (61, 84)	171 (151, 190)	311 (280, 342)	526 (475, 577)	822 (734, 910)
	**IUI/OS**						
	Breast (invasive)	32 (27, 37)	292 (270, 314)	905 (852, 957)	1832 (1732, 1931)	3011 (2854, 3167)	4404 (4170, 4638)
	Breast (in-situ)	4 (2, 7)	54 (39, 69)	168 (127, 208)	348 (270, 426)	592 (440, 743)	893 (620, 1165)
	Ovarian	5 (3, 6)	29 (21, 37)	78 (62, 94)	149 (120, 178)	251 (206, 296)	384 (313, 455)
	Ovarian (serous)	1 (0, 2)	9 (5, 13)	29 (20, 39)	64 (46, 82)	121 (90, 152)	209 (154, 263)
	Ovarian (non-serous)	2 (0, 5)	20 (10, 30)	37 (23, 51)	87 (62, 111)	140 (103, 177)	176 (127, 225)
	Uterine	3 (2, 5)	25 (19, 31)	80 (66, 95)	179 (149, 209)	352 (298, 407)	635 (540, 731)
	Thyroid	11 (8, 14)	84 (70, 99)	195 (167, 222)	323 (280, 365)	469 (408, 531)	631 (546, 717)
	Colorectal	7 (5, 9)	51 (42, 61)	139 (118, 160)	269 (231, 306)	471 (409, 533)	763 (662, 864)
	Melanoma (invasive)	42 (31, 52)	214 (184, 245)	442 (397, 487)	715 (651, 778)	1055 (963, 1146)	1451 (1316, 1586)
	Melanoma (in-situ)	25 (19, 32)	210 (178, 243)	612 (527, 698)	1249 (1066, 1432)	2083 (1711, 2453)	3091 (2434, 3744)
	Pancreatic	1 (0, 1)	5 (2, 7)	14 (8, 21)	31 (19, 43)	62 (39, 85)	120 (78, 162)
	Lung	1 (0, 1)	10 (7, 14)	38 (28, 47)	88 (68, 107)	178 (140, 217)	342 (269, 415)
	Haematological	13 (9, 16)	75 (62, 87)	179 (155, 203)	327 (286, 368)	560 (494, 626)	896 (786, 1006)
	**Clomiphene Citrate**						
	Breast (invasive)	31 (24, 38)	267 (243, 292)	782 (733, 831)	1578 (1482, 1675)	N/A	N/A

© 2026 Walker AR et al. *JAMA Network Open*. This is an open access article distributed under the terms of the CC-BY-NC-ND License, which does not permit alteration or commercial use, including those for text and data mining, AI training, and similar technologies.

	Breast (in-situ)	2 (2, 3)	26 (21, 31)	104 (87, 120)	252 (213, 291)	N/A	N/A
	Ovarian	4 (2, 5)	24 (18, 30)	55 (42, 67)	96 (74, 117)	N/A	N/A
	Ovarian (serous)	1 (0, 2)	8 (5, 12)	21 (13, 29)	40 (25, 54)	N/A	N/A
	Ovarian (non-serous)	3 (2, 4)	16 (11, 21)	34 (24, 43)	56 (40, 72)	N/A	N/A
	Uterine	8 (4, 11)	44 (34, 54)	110 (92, 127)	223 (188, 258)	N/A	N/A
	Thyroid	13 (11, 16)	104 (91, 116)	255 (227, 282)	421 (376, 466)	N/A	N/A
	Colorectal	8 (6, 10)	55 (46, 64)	141 (122, 161)	280 (242, 317)	N/A	N/A
	Melanoma (invasive)	25 (21, 29)	161 (146, 176)	379 (347, 412)	678 (619, 736)	N/A	N/A
	Melanoma (in-situ)	22 (19, 26)	162 (146, 177)	468 (428, 508)	1051 (959, 1142)	N/A	N/A
	Pancreatic cancer	0 (0, 1)	3 (1, 5)	11 (6, 16)	27 (15, 40)	N/A	N/A
	Lung	1 (0, 2)	11 (8, 14)	35 (25, 44)	94 (71, 117)	N/A	N/A
	Haematological	10 (8, 12)	68 (58, 78)	167 (146, 188)	307 (268, 347)	N/A	N/A
**Comparator**	**ART**						
	Breast (invasive)	31 (28, 34)	288 (275, 300)	885 (859, 910)	1766 (1721, 1812)	2879 (2811, 2947)	4193 (4082, 4303)
	Breast (in-situ)	4 (3, 5)	39 (34, 45)	145 (131, 159)	334 (303, 365)	611 (527, 695)	975 (794, 1156)
	Ovarian	2 (1, 3)	17 (14, 20)	50 (44, 56)	101 (90, 111)	177 (160, 194)	282 (251, 313)
	Ovarian (serous)	1 (0, 1)	7 (5, 9)	23 (19, 27)	50 (42, 57)	93 (81, 106)	156 (132, 180)
	Ovarian (non-serous)	2 (1, 2)	11 (9, 13)	27 (23, 32)	51 (44, 58)	83 (72, 94)	124 (105, 144)
	Uterine	2 (1, 3)	15 (12, 18)	52 (46, 57)	119 (107, 130)	246 (225, 266)	468 (425, 510)
	Thyroid	12 (10, 14)	80 (74, 87)	181 (169, 193)	303 (285, 321)	446 (420, 472)	606 (566, 645)
	Colorectal	7 (5, 8)	52 (47, 57)	140 (131, 150)	276 (258, 294)	484 (456, 512)	774 (723, 824)
	Melanoma (invasive)	23 (20, 26)	146 (136, 156)	333 (316, 349)	580 (555, 605)	901 (864, 938)	1286 (1223, 1349)
	Melanoma (in-situ)	26 (23, 30)	172 (160, 184)	468 (442, 493)	979 (916, 1043)	1675 (1520, 1830)	2536 (2236, 2835)
	Pancreatic	0 (0, 1)	4 (3, 6)	16 (13, 19)	38 (31, 44)	74 (63, 86)	129 (107, 150)
	Lung	2 (1, 2)	16 (13, 19)	55 (49, 61)	128 (116, 140)	265 (243, 286)	507 (463, 551)
	Haematological	7 (5, 8)	51 (46, 57)	138 (128, 148)	273 (255, 290)	488 (461, 516)	798 (746, 851)
	**IUI/OS**						
	Breast (invasive)	29 (25, 34)	268 (252, 285)	830 (797, 863)	1681 (1623, 1739)	2765 (2680, 2849)	4046 (3913, 4179)
	Breast (in-situ)	4 (2, 6)	46 (37, 56)	144 (124, 165)	299 (257, 342)	509 (404, 614)	768 (557, 978)
	Ovarian	3 (2, 4)	20 (15, 24)	52 (44, 61)	101 (87, 115)	169 (148, 190)	259 (224, 294)
	Ovarian (serous)	1 (0, 2)	6 (4, 9)	20 (15, 24)	43 (34, 52)	81 (66, 96)	140 (113, 167)
	Ovarian (non-serous)	2 (1, 4)	14 (10, 19)	33 (26, 41)	57 (46, 68)	86 (71, 101)	119 (96, 143)
	Uterine	2 (1, 3)	16 (12, 20)	52 (44, 60)	116 (102, 130)	229 (204, 253)	413 (366, 460)
	Thyroid	10 (7, 13)	76 (67, 86)	176 (160, 192)	291 (267, 315)	424 (391, 456)	570 (523, 618)
	Colorectal	7 (5, 9)	52 (44, 59)	140 (127, 153)	270 (247, 293)	474 (438, 509)	767 (705, 829)

© 2026 Walker AR et al. *JAMA Network Open*. This is an open access article distributed under the terms of the CC-BY-NC-ND License, which does not permit alteration or commercial use, including those for text and data mining, AI training, and similar technologies.

Melanoma (invasive)	25 (20, 30)	154 (140, 168)	343 (320, 365)	580 (546, 613)	883 (836, 930)	1246 (1172, 1319)
Melanoma (in-situ)	18 (14, 23)	151 (133, 168)	440 (400, 479)	898 (805, 990)	1499 (1279, 1718)	2227 (1809, 2644)
Pancreatic	1 (0, 2)	6 (3, 8)	17 (12, 22)	36 (28, 45)	73 (59, 87)	141 (112, 170)
Lung	1 (0, 2)	14 (11, 18)	52 (44, 61)	121 (106, 136)	246 (220, 273)	472 (421, 523)
Haematological	10 (8, 12)	59 (51, 67)	141 (127, 154)	257 (235, 280)	441 (407, 475)	706 (648, 763)
**Clomiphene Citrate**						
Breast (invasive)	22 (19, 25)	204 (192, 215)	678 (652, 704)	1514 (1462, 1566)	N/A	N/A
Breast (in-situ)	2 (1, 3)	22 (19, 26)	90 (81, 99)	218 (199, 238)	N/A	N/A
Ovarian	3 (2, 4)	17 (14, 21)	39 (33, 45)	68 (58, 79)	N/A	N/A
Ovarian (serous)	1 (0, 1)	6 (4, 8)	15 (12, 19)	29 (22, 36)	N/A	N/A
Ovarian (non-serous)	2 (1, 3)	11 (9, 14)	24 (19, 28)	39 (31, 47)	N/A	N/A
Uterine	1 (0, 2)	11 (8, 14)	34 (29, 40)	80 (67, 92)	N/A	N/A
Thyroid	10 (8, 12)	80 (73, 87)	197 (183, 210)	325 (303, 347)	N/A	N/A
Colorectal	7 (5, 9)	48 (42, 53)	122 (112, 133)	242 (222, 263)	N/A	N/A
Melanoma (invasive)	21 (18, 23)	132 (124, 141)	312 (295, 329)	557 (528, 587)	N/A	N/A
Melanoma (in-situ)	17 (15, 20)	125 (116, 134)	362 (342, 382)	814 (766, 861)	N/A	N/A
Pancreatic	0 (0, 1)	3 (2, 5)	12 (8, 15)	28 (21, 36)	N/A	N/A
Lung	1 (0, 2)	11 (9, 14)	36 (30, 42)	98 (85, 112)	N/A	N/A
Haematological	9 (7, 10)	58 (52, 63)	141 (130, 153)	260 (239, 280)	N/A	N/A

MAR = Medically Assisted Reproduction; ART=Assisted Reproductive Technology; IUI/OS=Intrauterine Insemination/Ovarian stimulation

© 2026 Walker AR et al. *JAMA Network Open*. This is an open access article distributed under the terms of the CC-BY-NC-ND License, which does not permit alteration or commercial use, including those for text and data mining, AI training, and similar technologies.

eTable 9: E-values over time for time-varying hazard ratio models.

	1 year	5 years	10 years	15 years	20 years	25 years
**ART**						
Ovarian	4.59 (2.86, 7.10)	2.79 (2.18, 3.50)	2.21 (1.70, 2.77)	1.94 (1.39, 2.53)	N/A[a]	N/A[a]
Ovarian (serous)	4.76 (2.27, 9.19)	2.64 (1.76, 3.73)	1.97 (1.23, 2.75)	N/A[a]	N/A[a]	N/A[a]
Uterine	4.24 (2.72, 6.41)	2.84 (2.24, 3.54)	2.31 (1.88, 2.78)	2.05 (1.62, 2.53)	1.89 (1.41, 2.40)	1.74 (1.13, 2.29)
Melanoma (invasive)	N/A[a]	1.96 (1.54, 2.39)	N/A[a]	N/A[a]	N/A[a]	N/A[a]
Pancreatic	4.20 (1.33, 10.16)	N/A[a]	N/A[a]	N/A[a]	N/A[a]	N/A[a]
Haematological	2.65 (1.84, 3.64)	1.72 (1.37, 2.08)	N/A[a]	N/A[a]	N/A[a]	N/A[a]
**IUI/OS**						
Melanoma (invasive)	2.37 (1.71, 3.13)	1.83 (1.52, 2.14)	1.62 (1.29, 1.93)	1.53 (1.11, 1.88)	N/A[a]	N/A[a]
Ovarian (non-serous)	N/A[a]	N/A[a]	N/A[a]	4.16 (2.42, 6.78)	N/A[a]	N/A[a]
**Clomiphene Citrate**						
Breast cancer (invasive)	2.28 (1.79, 2.82)	1.69 (1.44, 1.93)	N/A[a]	N/A[a]	N/A	N/A
Uterine	9.60 (5.32, 16.95)	5.73 (4.51, 7.24)	4.76 (3.62, 6.18)	4.24 (2.99, 5.89)	N/A	N/A

[a]E-value not calculated for results where hazard ratio confidence interval includes 1

ART=Assisted Reproductive Technology; IUI/OS=Intrauterine Insemination/Ovarian Stimulation

© 2026 Walker AR et al. *JAMA Network Open*. This is an open access article distributed under the terms of the CC-BY-NC-ND License, which does not permit alteration or commercial use, including those for text and data mining, AI training, and similar technologies.

eTable 10: Deviance and likelihood ratio tests for selection of models (No prior cancer sensitivity analysis).

MAR treatment type	Deviance difference from no time-varying			Likelihood ratio test to non time varying			Deviance difference from 0 spline		Likelihood ratio test to 0 spline		Deviance difference from 1 spline	Likelihood ratio test to 1 spline	
Cancer type	0 splines	1 spline	3 splines	0 splines	1 spline	3 splines	1 spline	3 splines	1 spline	3 splines	3 splines	3 splines	Final model
**ART**													
Breast (invasive)	1.92	2.30	3.65	0.166	0.317	0.455	0.37	1.73	0.540	0.630	1.36	0.508	Non-time varying
Breast (in-situ)	1.11	1.11	1.74	0.292	0.574	0.784	0.00	0.63	0.990	0.890	0.63	0.731	Non-time varying
Ovarian	7.74	9.19	11.78	0.005	0.010	0.019	1.45	4.04	0.229	0.257	2.59	0.274	0 spline
Ovarian (serous)	5.01	7.07	9.41	0.025	0.029	0.052	2.06	4.40	0.151	0.222	2.34	0.311	0 spline
Ovarian (non-serous)	2.70	2.76	5.28	0.100	0.251	0.260	0.06	2.58	0.805	0.461	2.52	0.284	Non-time varying
Uterine	7.68	10.34	11.33	0.006	0.006	0.023	2.66	3.65	0.103	0.302	0.99	0.609	0 spline
Thyroid	0.00	0.00	0.83	0.945	0.998	0.935	0.00	0.82	0.999	0.844	0.82	0.663	Non-time varying
Colorectal	2.66	2.74	3.20	0.103	0.255	0.526	0.07	0.53	0.789	0.912	0.46	0.794	Non-time varying
Melanoma (invasive)	4.29	4.93	10.63	0.038	0.085	0.031	0.64	6.34	0.424	0.096	5.70	0.058	0 spline
Melanoma (in-situ)	0.01	1.77	1.97	0.921	0.412	0.742	1.76	1.96	0.184	0.581	0.19	0.907	Non-time varying
Pancreatic	7.18	7.28	9.69	0.007	0.026	0.046	0.10	2.51	0.752	0.473	2.41	0.299	0 spline
Lung	0.08	0.82	2.40	0.773	0.665	0.662	0.73	2.32	0.392	0.509	1.58	0.453	Non-time varying
Haematological	8.96	9.51	9.66	0.003	0.009	0.047	0.55	0.70	0.457	0.874	0.14	0.931	0 spline
**IUI/OS**													
Breast (invasive)	1.33	1.89	5.16	0.248	0.388	0.272	0.56	3.82	0.454	0.281	3.26	0.196	Non-time varying
Breast (in-situ)	1.07	2.27	4.98	0.302	0.322	0.289	1.20	3.91	0.273	0.271	2.71	0.258	Non-time varying
Ovarian	0.00	0.32	0.41	0.959	0.854	0.981	0.31	0.41	0.576	0.938	0.10	0.952	Non-time varying
Ovarian (serous)	1.21	1.92	NA	0.272	0.384	NA	0.71	NA	0.400	NA	NA	NA	Non-time varying
Ovarian (non-serous)	0.69	0.70	9.37	0.407	0.703	0.052	0.02	8.68	0.901	0.034	8.67	0.013	Non-time varying
Uterine	0.54	0.74	3.31	0.461	0.692	0.508	0.19	2.76	0.661	0.430	2.57	0.277	Non-time varying
Thyroid	0.03	0.04	5.27	0.860	0.979	0.261	0.01	5.23	0.913	0.155	5.22	0.073	Non-time varying
Colorectal	2.63	2.64	3.33	0.105	0.268	0.504	0.00	0.70	0.982	0.874	0.70	0.706	Non-time varying
Melanoma (invasive)	4.43	5.53	9.01	0.035	0.063	0.061	1.09	4.58	0.296	0.205	3.49	0.175	0 spline
Melanoma (in-situ)	0.84	1.50	3.78	0.359	0.472	0.436	0.66	2.94	0.416	0.401	2.28	0.320	Non-time varying
Pancreatic	0.09	1.63	NA	0.767	0.442	NA	1.55	NA	0.214	NA	NA	NA	Non-time varying
Lung	0.02	0.43	0.89	0.898	0.808	0.927	0.41	0.87	0.521	0.833	0.46	0.795	Non-time varying

© 2026 Walker AR et al. *JAMA Network Open*. This is an open access article distributed under the terms of the CC-BY-NC-ND License, which does not permit alteration or commercial use, including those for text and data mining, AI training, and similar technologies.

Haematological	1.46	1.58	8.86	0.227	0.454	0.065	0.12	7.40	0.734	0.060	7.28	0.026	Non-time varying
**Clomiphene Citrate**													
Breast (invasive)	12.55	15.81	17.04	0.000	0.000	0.002	3.27	4.49	0.071	0.213	1.23	0.542	0 spline
Breast (in-situ)	0.66	0.74	1.07	0.417	0.692	0.900	0.08	0.41	0.781	0.939	0.33	0.849	Non-time varying
Ovarian	1.48	1.97	4.56	0.224	0.373	0.336	0.50	3.08	0.481	0.379	2.59	0.274	Non-time varying
Ovarian (serous)	0.01	3.50	5.97	0.914	0.174	0.202	3.49	NA	0.062	NA	NA	NA	Non-time varying
Ovarian (non-serous)	1.86	2.07	4.98	0.173	0.356	0.289	0.21	NA	0.645	NA	NA	NA	Non-time varying
Uterine	6.56	7.13	10.65	0.010	0.028	0.031	0.57	4.09	0.450	0.252	3.52	0.172	0 spline
Thyroid	0.73	0.90	1.69	0.391	0.638	0.793	0.16	0.95	0.685	0.813	0.79	0.675	Non-time varying
Colorectal	2.12	4.21	5.81	0.145	0.122	0.214	2.09	3.69	0.148	0.297	1.60	0.450	Non-time varying
Melanoma (invasive)	0.22	0.23	5.05	0.635	0.893	0.282	0.00	4.83	0.971	0.185	4.83	0.089	Non-time varying
Melanoma (in-situ)	0.74	4.56	5.57	0.388	0.102	0.234	3.82	4.83	0.051	0.185	1.01	0.603	Non-time varying
Pancreatic	0.16	0.16	1.91	0.690	0.924	0.751	0.00	1.76	0.992	0.625	1.76	0.416	Non-time varying
Lung	0.62	0.78	2.59	0.430	0.677	0.629	0.16	1.96	0.693	0.580	1.81	0.405	Non-time varying
Haematological	0.44	3.08	7.96	0.505	0.214	0.093	2.64	7.51	0.104	0.057	4.87	0.088	Non-time varying

MAR= Medically Assisted Reproduction; ART=Assisted Reproductive Technology; IUI/OS= Intrauterine Insemination/Ovarian Stimulation. NA indicates model did not fit

© 2026 Walker AR et al. *JAMA Network Open*. This is an open access article distributed under the terms of the CC-BY-NC-ND License, which does not permit alteration or commercial use, including those for text and data mining, AI training, and similar technologies.

eTable 11: Hazard ratios, E-values, and cumulative marginal difference in incident cancers (per 100,000 women) for each emulated target trial (No prior cancer sensitivity analysis).

MAR treatment type	Hazard ratio			Cumulative marginal difference in incident cancers per 100,000 women					
Cancer type	Estimate (95% CI)	Standard error	E-Value (95% CI)	1 year	5 years	10 years	15 years	20 years	25 years
**ART**									
Breast (invasive)	1.11 (1.06, 1.16)	0.03	1.46 (1.31, 1.59)	3 (2, 5)	31 (17, 45)	95 (51, 138)	189 (102, 275)	306 (166, 447)	443 (240, 646)
Breast (in-situ)	1.29 (1.09, 1.52)	0.11	1.90 (1.42, 2.40)	1 (0, 2)	12 (4, 20)	42 (13, 71)	96 (30, 162)	175 (53, 296)	277 (82, 472)
Ovarian	Time varying			4 (2, 7)	18 (10, 27)	35 (21, 49)	52 (29, 75)	72 (33, 110)	91 (28, 154)
Ovarian (serous)	Time varying			2 (0, 3)	8 (3, 13)	14 (5, 23)	20 (4, 36)	26 (-1, 52)	28 (-17, 74)
Ovarian (non-serous)	1.61 (1.28, 2.02)	0.19	2.60 (1.88, 3.45)	1 (0, 2)	7 (3, 11)	17 (8, 26)	31 (14, 48)	51 (24, 78)	76 (35, 117)
Uterine	Time varying			4 (2, 6)	16 (9, 23)	37 (23, 50)	63 (39, 88)	104 (58, 149)	159 (72, 247)
Thyroid	1.14 (1.02, 1.28)	0.06	1.55 (1.18, 1.88)	2 (0, 3)	11 (2, 21)	26 (4, 48)	43 (6, 80)	64 (9, 118)	86 (13, 160)
Colorectal	1.12 (1.00, 1.25)	0.06	*	1 (0, 2)	6 (0, 12)	16 (-1, 33)	32 (-1, 65)	56 (-2, 114)	89 (-3, 182)
Melanoma (invasive)	Time varying			9 (2, 15)	33 (13, 53)	53 (20, 85)	69 (19, 119)	84 (5, 162)	93 (-25, 211)
Melanoma (in-situ)	1.15 (1.04, 1.28)	0.06	1.58 (1.24, 1.89)	4 (1, 7)	26 (6, 46)	71 (16, 126)	150 (34, 266)	258 (58, 457)	390 (87, 694)
Pancreatic	Time varying			1 (0, 3)	3 (-1, 7)	4 (-4, 11)	-1 (-12, 11)	-12 (-32, 9)	-32 (-68, 3)
Lung	0.84 (0.71, 1.00)	0.07	*	0 (-1, 0)	-2 (-5, 0)	-9 (-17, -1)	-20 (-39, -1)	-42 (-80, -3)	-80 (-153, -6)
Haematological cancer	Time varying			6 (2, 10)	21 (8, 33)	31 (11, 52)	37 (4, 70)	36 (-22, 94)	23 (-75, 121)
**IUI/OS**									
Breast (invasive)	1.10 (1.05, 1.14)	0.02	1.42 (1.29, 1.54)	10 (6, 15)	75 (42, 109)	197 (110, 284)	364 (203, 524)	585 (327, 843)	853 (477, 1229)
Breast (in-situ)	1.09 (1.03, 1.16)	0.03	1.40 (1.19, 1.59)	3 (1, 5)	24 (7, 41)	74 (21, 127)	150 (42, 257)	245 (69, 421)	355 (100, 611)
Ovarian	1.17 (0.91, 1.52)	0.16	N/A[a]	1 (-1, 2)	8 (-6, 22)	25 (-17, 67)	52 (-35, 139)	89 (-60, 238)	135 (-90, 361)
Ovarian (serous)	1.47 (1.18, 1.82)	0.16	2.29 (1.64, 3.05)	1 (0, 2)	9 (3, 15)	24 (9, 39)	46 (17, 75)	78 (29, 127)	120 (45, 196)
Ovarian (non-serous)	1.52 (1.11, 2.08)	0.24	2.40 (1.45, 3.57)	0 (0, 1)	3 (0, 6)	10 (1, 18)	21 (3, 39)	41 (7, 74)	71 (12, 130)
Uterine	1.42 (1.05, 1.93)	0.22	2.20 (1.28, 3.26)	0 (-3, 3)	4 (-6, 15)	1 (-14, 17)	26 (-1, 52)	50 (11, 90)	53 (-1, 107)
Thyroid	1.53 (1.28, 1.83)	0.14	2.43 (1.87, 3.07)	1 (0, 2)	9 (4, 13)	28 (14, 41)	62 (32, 92)	121 (64, 179)	218 (115, 320)
Colorectal	1.11 (0.95, 1.29)	0.09	N/A[a]	1 (-1, 3)	8 (-4, 21)	19 (-10, 48)	31 (-16, 79)	45 (-24, 114)	61 (-32, 154)
Melanoma (invasive)	Time varying			17 (5, 28)	59 (28, 91)	98 (50, 146)	133 (64, 202)	169 (67, 271)	202 (53, 351)
Melanoma (in-situ)	1.38 (1.18, 1.63)	0.11	2.11 (1.63, 2.64)	7 (3, 11)	58 (26, 89)	168 (76, 260)	343 (155, 530)	571 (254, 888)	846 (368, 1324)
Pancreatic	0.87 (0.58, 1.31)	0.18	N/A[a]	0 (0, 0)	-1 (-3, 1)	-2 (-8, 4)	-4 (-17, 8)	-9 (-35, 17)	-17 (-65, 32)

© 2026 Walker AR et al. *JAMA Network Open*. This is an open access article distributed under the terms of the CC-BY-NC-ND License, which does not permit alteration or commercial use, including those for text and data mining, AI training, and similar technologies.

Lung	0.73 (0.58, 0.92)	0.09	N/A[a]	0 (-1, 0)	-4 (-6, -1)	-14 (-23, -4)	-32 (-54, -10)	-66 (-110, -21)	-124 (-209, -40)
Haematological cancer	1.28 (1.11, 1.47)	0.09	1.87 (1.46, 2.30)	3 (1, 5)	16 (6, 26)	38 (15, 62)	70 (27, 113)	121 (47, 194)	194 (76, 313)
**Clomiphene Citrate**									
Breast (invasive)		Time varying		13 (6, 19)	52 (28, 77)	85 (34, 135)	82 (-25, 190)	N/A	N/A
Breast (in-situ)	1.16 (0.97, 1.38)	0.10	N/A[a]	0 (0, 1)	3 (-1, 8)	14 (-4, 32)	34 (-9, 77)	N/A	N/A
Ovarian	1.39 (1.07, 1.82)	0.19	2.14 (1.34, 3.04)	1 (0, 2)	7 (1, 13)	15 (2, 28)	27 (4, 49)	N/A	N/A
Ovarian (serous)	1.42 (0.93, 2.15)	0.30	N/A[a]	0 (0, 1)	2 (-1, 6)	6 (-2, 14)	12 (-4, 27)	N/A	N/A
Ovarian (non-serous)	1.38 (0.98, 1.95)	0.24	N/A[a]	1 (0, 2)	4 (-1, 9)	9 (-1, 19)	15 (-2, 32)	N/A	N/A
Uterine		Time varying		6 (3, 10)	33 (23, 43)	74 (56, 91)	140 (104, 176)	N/A	N/A
Thyroid	1.32 (1.16, 1.49)	0.08	1.96 (1.60, 2.34)	3 (2, 5)	25 (13, 37)	62 (32, 91)	102 (53, 151)	N/A	N/A
Colorectal	1.18 (1.01, 1.38)	0.09	1.64 (1.11, 2.09)	1 (0, 2)	8 (0, 17)	22 (0, 43)	43 (1, 84)	N/A	N/A
Melanoma (invasive)	1.21 (1.10, 1.34)	0.06	1.72 (1.43, 2.01)	4 (2, 7)	28 (13, 42)	65 (30, 100)	117 (54, 180)	N/A	N/A
Melanoma (in-situ)	1.30 (1.18, 1.44)	0.06	1.93 (1.65, 2.23)	5 (3, 7)	38 (23, 53)	109 (66, 152)	244 (148, 340)	N/A	N/A
Pancreatic	0.98 (0.58, 1.64)	0.26	N/A[a]	0 (0, 0)	0 (-2, 2)	0 (-6, 6)	-1 (-15, 13)	N/A	N/A
Lung	0.94 (0.71, 1.26)	0.14	N/A[a]	0 (0, 0)	-1 (-4, 2)	-2 (-12, 8)	-5 (-32, 21)	N/A	N/A
Haematological cancer	1.20 (1.03, 1.38)	0.09	1.68 (1.22, 2.11)	2 (0, 3)	11 (2, 20)	27 (4, 50)	50 (7, 93)	N/A	N/A

[a]E-value not calculated for results where hazard ratio confidence interval is entirely below or includes 1

MAR = Medically Assisted Reproduction; ART=Assisted Reproductive Technology; IUI/OS=Intrauterine Insemination/Ovarian Stimulation

© 2026 Walker AR et al. *JAMA Network Open*. This is an open access article distributed under the terms of the CC-BY-NC-ND License, which does not permit alteration or commercial use, including those for text and data mining, AI training, and similar technologies.

eTable 12: E-values over time for time-varying hazard ratio models (No prior cancer sensitivity analysis).

	1 year	5 years	10 years	15 years	20 years	25 years
**ART**						
Ovarian	1.86 (1.65, 2.08)	1.52 (1.42, 1.62)	1.37 (1.26, 1.47)	1.28 (1.14, 1.40)	N/A[a]	N/A[a]
Ovarian (serous)	4.39 (2.72, 6.82)	2.71 (2.11, 3.41)	2.16 (1.66, 2.72)	1.91 (1.35, 2.50)	N/A[a]	N/A[a]
Uterine	4.60 (2.16, 8.96)	2.58 (1.70, 3.67)	1.93 (1.17, 2.71)	N/A[a]	N/A[a]	N/A[a]
Melanoma (invasive)	4.25 (2.69, 6.47)	2.84 (2.23, 3.56)	2.31 (1.88, 2.79)	2.06 (1.62, 2.53)	1.89 (1.41, 2.40)	1.74 (1.14, 2.30)
Pancreatic	1.94 (1.45, 2.47)	1.54 (1.28, 1.78)	N/A[a]	N/A[a]	N/A[a]	N/A[a]
Haematological	4.45 (1.42, 10.94)	N/A[a]	N/A[a]	N/A[a]	N/A[a]	N/A[a]
**IUI/OS**	2.60 (1.78, 3.60)	1.71 (1.34, 2.07)	N/A[a]	N/A[a]	N/A[a]	N/A[a]
Melanoma (invasive)						
Clomiphene Citrate	2.36 (1.70, 3.12)	1.82 (1.51, 2.14)	1.62 (1.29, 1.93)	1.53 (1.10, 1.88)	N/A[a]	N/A[a]
Breast cancer (invasive)						
Uterine	2.02 (1.75, 2.29)	1.70 (1.56, 1.84)	1.42 (1.25, 1.58)	N/A[a]	N/A	N/A

[a]E-value not calculated for results where hazard ratio confidence interval includes 1

ART=Assisted Reproductive Technology; IUI/OS=Intrauterine Insemination/Ovarian Stimulation

© 2026 Walker AR et al. *JAMA Network Open*. This is an open access article distributed under the terms of the CC-BY-NC-ND License, which does not permit alteration or commercial use, including those for text and data mining, AI training, and similar technologies.
