# Supplementary material for: Medically Assisted Reproduction and Hormone-Related Cancers
Source: JAMA Netw Open. 2026 Jul 13;9(7):e2622832. doi: 10.1001/jamanetworkopen.2026.22832 (PMC13366193; doi:10.1001/jamanetworkopen.2026.22832)
Supplement: Supplement 2. — Data Sharing Statement [file jamanetwopen-e2622832-s002.pdf]

Data Sharing Statement

Walker. Medically Assisted Reproduction and Hormone-Related Cancers. *JAMA Netw Open*. Published July 13, 2026. doi:10.1001/jamanetworkopen.2026.22832

Data
Data available: No

Additional Information
Explanation for why data not available: The analyses were based on data from different registries and administrative datasets (Australian Department of Health and Aged Care, State and Territory Health Departments, and the Australian Institute of Health and Welfare). And the data can be made available on request to each of the data custodians after ethical approval from the relevant Human Research Ethics Committees.
